# Immunomodulation in the Treatment of Periodontitis: Progress and Perspectives

**DOI:** 10.3389/fimmu.2021.781378

**Published:** 2021-11-19

**Authors:** Bo Yang, Xuefei Pang, Zhipeng Li, Zhuofan Chen, Yan Wang

**Affiliations:** ^1^ Hospital of Stomatology, Sun Yat-sen University, Guangzhou, China; ^2^ Guangdong Provincial Key Laboratory of Stomatology, Guangzhou, China; ^3^ Guanghua School of Stomatology, Sun Yat-sen University, Guangzhou, China

**Keywords:** periodontitis, immune microenvironment (IME), immunomodulation, drug therapy, microbial therapy, stem cell therapy, gene therapy

## Abstract

Periodontitis is one of the most common dental diseases. Compared with healthy periodontal tissues, the immune microenvironment plays the key role in periodontitis by allowing the invasion of pathogens. It is possible that modulating the immune microenvironment can supplement traditional treatments and may even promote periodontal regeneration by using stem cells, bacteria, etc. New anti-inflammatory therapies can enhance the generation of a viable local immune microenvironment and promote cell homing and tissue formation, thereby achieving higher levels of immune regulation and tissue repair. We screened recent studies to summarize the advances of the immunomodulatory treatments for periodontitis in the aspects of drug therapy, microbial therapy, stem cell therapy, gene therapy and other therapies. In addition, we included the changes of immune cells and cytokines in the immune microenvironment of periodontitis in the section of drug therapy so as to make it clearer how the treatments took effects accordingly. In the future, more research needs to be done to improve immunotherapy methods and understand the risks and long-term efficacy of these methods in periodontitis.

## Introduction

Periodontitis, one of the most common dental diseases, is caused by an inflammatory process affecting periodontal tissues and is indicated by periodontal soft tissue inflammation and the progressive loss of periodontal ligaments and alveolar bone (1). Furthermore, severe periodontitis can lead to facial collapse, impaired mastication, and effects on the digestive system. It is also associated with various systemic and distal inflammatory diseases, including diabetes, cardiovascular disease, rheumatoid arthritis, metabolic syndrome, and Alzheimer’s disease (2–5).

The etiology and mechanism of periodontitis is extremely complex. The occurrence and development of periodontitis is the result of the interaction between bacteria and the host (6). Periodontal tissue destruction begins with an inflammatory process caused by oral bacterial infection (2). Host susceptibility is a decisive factor in the development of periodontitis. The human oral cavity is a complex ecological environment, and teeth, gingival crevices and other parts are conducive to bacteria attachment and reproduction (2). Host susceptibility and the effects of oral bacteria lead to the destruction of periodontal tissues, eventually resulting in loosening and loss of the teeth if untreated (7).

Periodontitis initiation and progression are related to multiple etiologic and risk factors. However, the most critical in periodontal disease pathogenesis is a reciprocally reinforced interplay between microbial dysbiosis and destructive inflammation (8). Pathogens induce periodontitis in susceptible patients and most of the time, the immune system is very efficient and prevents disease progression until the microbial dysbiotic environment has been established. A complex microbial community is involved in periodontitis pathogenesis (9), and *Porphyromonas gingivalis (P. gingivalis*), *Treponema denticola*, and *Tannerella forsythia* are most often found (10–16). Pathogens can affect periodontal tissue cells by regulating the immune system (17) and leukocytes are essential players in periodontitis by control of gingival biofilm pathogenicity, activation of adaptive immunity, as well as non-resolving inflammation and collateral tissue damage (18).

Periodontitis should be treated as early as possible. Mild to moderate cases can usually be managed by nonsurgical treatments, including auxiliary antibiotics, scaling, and root planning (19, 20). For severe cases that cannot be fully controlled by nonsurgical treatments, surgical methods can reduce pocket depth and generate anatomical contours at the periodontal interface (21). However, the treatment for periodontitis is not ideal. Even in patients receiving regular professional interventions, periodontitis continues to progress and teeth are lost (22). Moreover, because the cost of treatment is high, periodontitis is still a major public health and economic burden (8).

**Figure 1:**
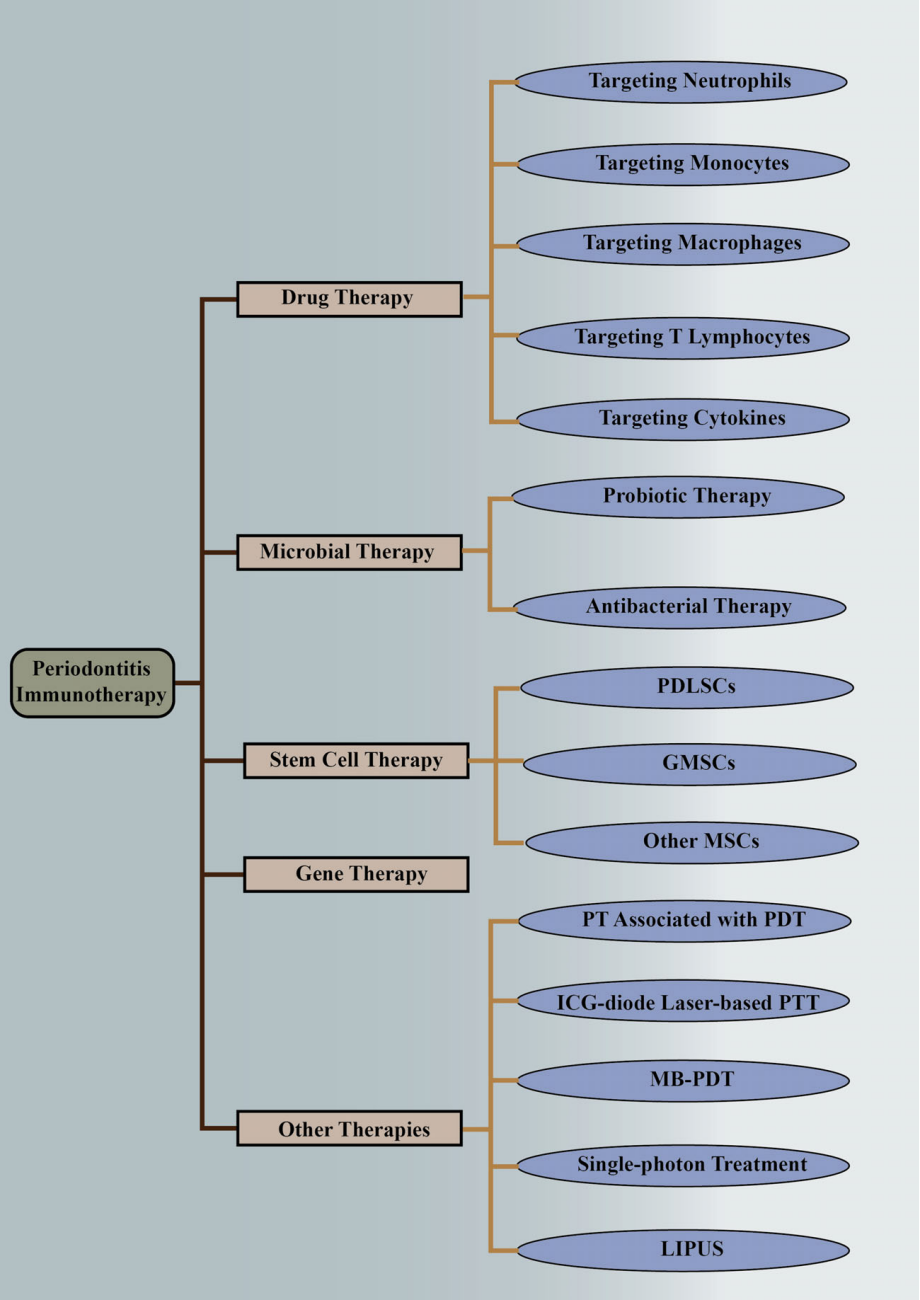
Immunotherapies of periodontitis. The above is a detailed classification of periodontitis immunotherapy, including drug therapy, microbial therapy, stem cell therapy, gene therapy, and other therapies. They are followed by more detailed classifications. PDLSCs, periodontal ligament stem cells; GMSCs, gingival mesenchymal stem cells; MSCs, mesenchymal stem cells; PT, periodontal therapy; PDT, photodynamic therapy; ICG, indocyanine green; PTT, photothermal therapy; MB-PDT, methylene blue-mediated photodynamic therapy; LIPUS, low-intensity pulsed ultrasound.

Recently, much attention has been drawn to regulating the immune response to putative periodontal pathogens in order to resolve inflammation, control the osteolytic environment, and restore physiological bone formation (18). Drugs, stem cells, and other therapies targeting the immune microenvironment have shown promising applications. In this study, we systematically reviewed the applications of immune modulation in the treatment of periodontitis, especially those targeting the immune microenvironment changes in periodontitis **(** **Figure 1** **)**.

## Drug Therapy

### Drugs Targeting Neutrophils

As the host’s first line of defense against pathogenic microorganisms (18, 23, 24), neutrophil homeostasis is key to periodontal health (1).

The periodontal lesion is initiated as acute inflammation characterized by increased numbers of neutrophils migrating into the gingival crevice through the junctional epithelium (1, 25) as a result of chemotaxis by plaque. They are activated by chemoattractants macrophage inflammatory protein-1α (MIP-1α), C-X-C motif ligand 8 (CXCL8) and constitutive higher reactive oxygen species (ROS) (26) and initiate phagocytosis with the assistance of antibodies and complement (27, 28), causing tissue damage (29) and excessive release of destructive molecules, which can be used to distinguish healthy and inflammatory periodontal tissues (30). In patients with periodontitis, recruitment, migration, and infiltration of neutrophils are increased in the early stage, while a significant reduction in phagocyte functions of neutrophils was observed in individuals with periodontitis (31). All of these changes are influenced by cytokines [e.g., granulocyte-colony stimulating factor (G-CSF)] (25), miRNAs [e.g., nod-like receptor 12 (NLRP12)] (32) and inflammasomes [e.g., nod-like receptor 12(NLRP12)] (33). Proinflammatory cytokines [e.g., tumor necrosis factor (TNF)-α and interleukin (IL)-8], neutrophil enzymes, eosinophil cationic protein (ECP), histidine decarboxylase, histamine and neutrophil elastase (NE) secreted by neutrophils are increased (34–37) and anti-inflammatory cytokines such as IL-10 are decreased (38). All of these changes are affected by bacteria (such as *P. gingivalis*) and bacterial products [such as leukocyte toxin (LtxA)] (36, 38).

Oxidative stress is considered to be an important component in various diseases (39). Polyphenols are now attracting attention as potential sources of agents that can inhibit, reverse, or delay the progression of diseases caused by oxidative stress and inflammatory processes. The highest concentration of active polyphenols has been found in the oral mucosa (40). Resveratrol, quercetin, and N-acetylcysteine (NAC) can reduce the production of ROS by neutrophils and upregulate the synthesis of the type 1 collagen gene, therefore contributing to the integrity of gingival tissues and prevention of periodontitis. Among the three, resveratrol has the best effect as an antioxidant that slows the progression of periodontitis. However, further studies using *in vivo* models are necessary to support the clinical use of antioxidants as a supplement to reduce oxidative stress and prevent periodontitis in humans (41).

**Figure 2:**
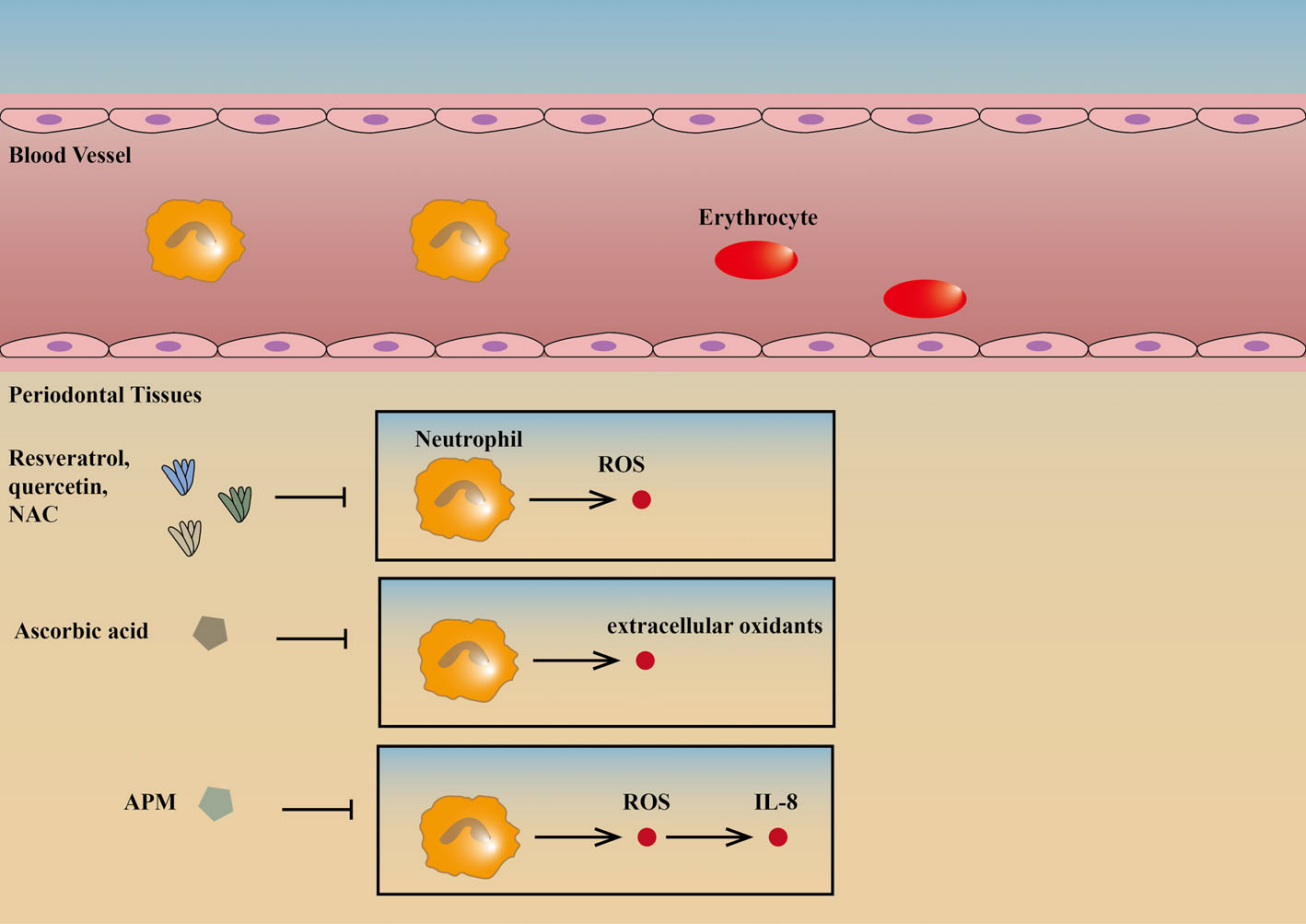
Drugs targeting neutrophils. Resveratrol, quercetin, and NAC can reduce the production of ROS by neutrophils, therefore contributing to the integrity of gingival tissues and prevention of periodontitis (41). Ascorbic acid can reduce inflammation in patients with periodontitis possibly because it usually acts as a reducing agent and can be used to treat periodontitis by reducing the extracellular oxidants of neutrophils (42, 43). APM is effective in inhibiting IL-8 production and decreasing cell damage through the suppression of intracellular ROS (44). ↑ is a symbol for positive effects, and ↓ is a symbol for negative effects and the same goes for the figures and tables below. NAC, N-acetylcysteine; ROS, reactive oxygen species; APM, L-Ascorbic acid 2-phosphate magnesium salt.

Progress have been made in the treatment of periodontitis with vitamins targeting neutrophils. Clinical studies have shown that ascorbic acid (vitamin C) can reduce inflammation in patients with periodontitis possibly because it usually acts as a reducing agent and can be used to treat periodontitis by reducing the extracellular oxidants of neutrophils (42, 43). An L-ascorbic acid derivative, L-Ascorbic acid 2-phosphate magnesium salt (APM), is effective in decreasing cell damage through the suppression of H_2_O_2_-induced intracellular ROS and inhibited IL-8 production through the suppression of TNF-α-induced intracellular ROS. This suggests that local application of APM can help to prevent periodontal diseases (44). In addition, 1, 25 dihydroxivitamin D3 can promote neutrophil apoptosis in type 2 diabetic periodontitis through the P38/MAPK pathway, reducing periodontitis (45) (**Figure 2** **)**.

However, the approach of using small molecules based on the mechanism of oxidative stress using exogenous antioxidants such as vitamin C to treat other inflammation-related diseases has failed. Therefore, the prospects for these treatments are not very optimistic. Periodontitis may result from interference at the ROS level, and the future research focuses on disease-related ROS source-specific inhibition (46). In this regard, resveratrol has the greater advantage (44).

### Drugs Targeting Monocytes

Monocytes, an important cellular defense system against pathogens, significantly increase in periodontitis tissues, especially intermediate monocytes (47). A significantly higher proportion of intermediate [Cluster of Differentiation (CD)14(+)CD16(+)] monocytes was observed in chronic periodontitis and they overexpressed human leukocyte antigen-DR (HLA-DR) and programmed cell death ligand 1 (PD-L1), indicating an activated inflammatory state (48). In addition, CD45RA(+) monocytes were increased in aggressive periodontitis (47). Depressed chemotaxis of monocyte results in increased periodontal destruction (49). There is also a reduction in the function of phagocytes of monocytes, suggesting a decrease in immune defenses in periodontitis (31). Pathogens stimulation of monocytes resulted in increased CD40 and CD54 expression, and enhanced the secretion of high levels of cytokines such as TNF-α, IL-1β, IL-6, IL-8, IL-17, IL-23, monocyte chemoattractant protein-1 (MCP-1) and interferon inducible protein-10 (50, 51).

Resveratrol can treat periodontitis by reducing *P. gingivalis*-mediated activation of the NF-κB signaling pathway. The effect on NF-κB activation likely results from the ability of resveratrol to act as a proliferator-activated receptor-γ (PPAR-γ) agonist. It can also attenuate triggering receptor expressed on myeloid cells-1(TREM-1) gene expression as well as soluble TREM-1 secretion in monocytes (52).

In addition, intracanal metformin for apical periodontitis has therapeutic efficacy. It can suppress lipopolysaccharide (LPS)-induced inducible nitric oxide synthase (iNOS) and NO production by monocytes, therefore inhibiting LPS-enhanced chemokine (C-C motif) ligand 2 (CCL-2) synthesis (53). Both of these drugs can eventually reduce bone resorption and improve periodontitis.

### Drugs Targeting Macrophages

Macrophages are central players in the destructive and reparative phases of periodontal disease (54). The behavior changes of macrophages are closely related to the pathogens. Pro-inflammatory macrophages increase and are activated in periodontitis (55). Increased proinflammatory responses, phagocytosis, and metabolic activity of macrophages in diseased periodontal tissue are mainly affected by various pathogenic bacteria such as *Fecal coliforms* and bacterial products such as LtxA (56, 57). Polarization is the main feature of macrophages in periodontitis that differentiates them from those in normal tissues (58). In periodontitis, macrophages tend to differentiate in the direction of M1, while M2 differentiation is inhibited significantly (48). Periodontitis is characterized by increased production of proinflammatory mediators and matrix-degrading enzymes by macrophages and increased osteoclastic activity (55). In addition, macrophages secrete increased proinflammatory cytokines such as TNF-α, interferon-γ (IFN-γ), IL-1α, IL-1β, IL-6, and IL-12; increased adhesion factors such as CXCL5 and CXCL1 (54, 59, 60); and increased inflammatory bodies such as NLRP3. In addition, the expression of other molecules such as toll-like receptors 2 (TLR2), TLR4, and nucleotide-binding oligomerization domain 2 (NOD2) is also increased (61), mainly by *in vivo* cytokines such as IL-17 and pathogenic bacteria such as *Aggregatibacter actinomycetemcomitans (A. actinomycetemcomitans)* (62, 63).

There is much research on drugs targeting macrophages to treat periodontitis. Proanthocyanidins (PACN) and cranberry proanthocyanidins (PACs) are two of the most active substances with positive effects on both cell behavior and molecular expression. Because of a lower risk of development of resistance and side effects, PACN, a multicomponent plant-derived antibacterial substance, has become a promising alternative and adjunctive therapy candidate for the treatment of periodontitis. Pelargonium sidoides dendritic cell root extract (PSRE), as one of the most PACN-enriched plants, can inhibit IL-8 and prostaglandin E2 (PGE2) produced by LPS-induced fibroblasts and IL-6 by leukocytes, blocking the expression of CD80 and CD86 on the surface of macrophages and IL-1 and cyclooxygenase-2 (COX-2) in leukocytes. Thus, PACN could be an effective drug for periodontitis (64).

**Figure 3:**
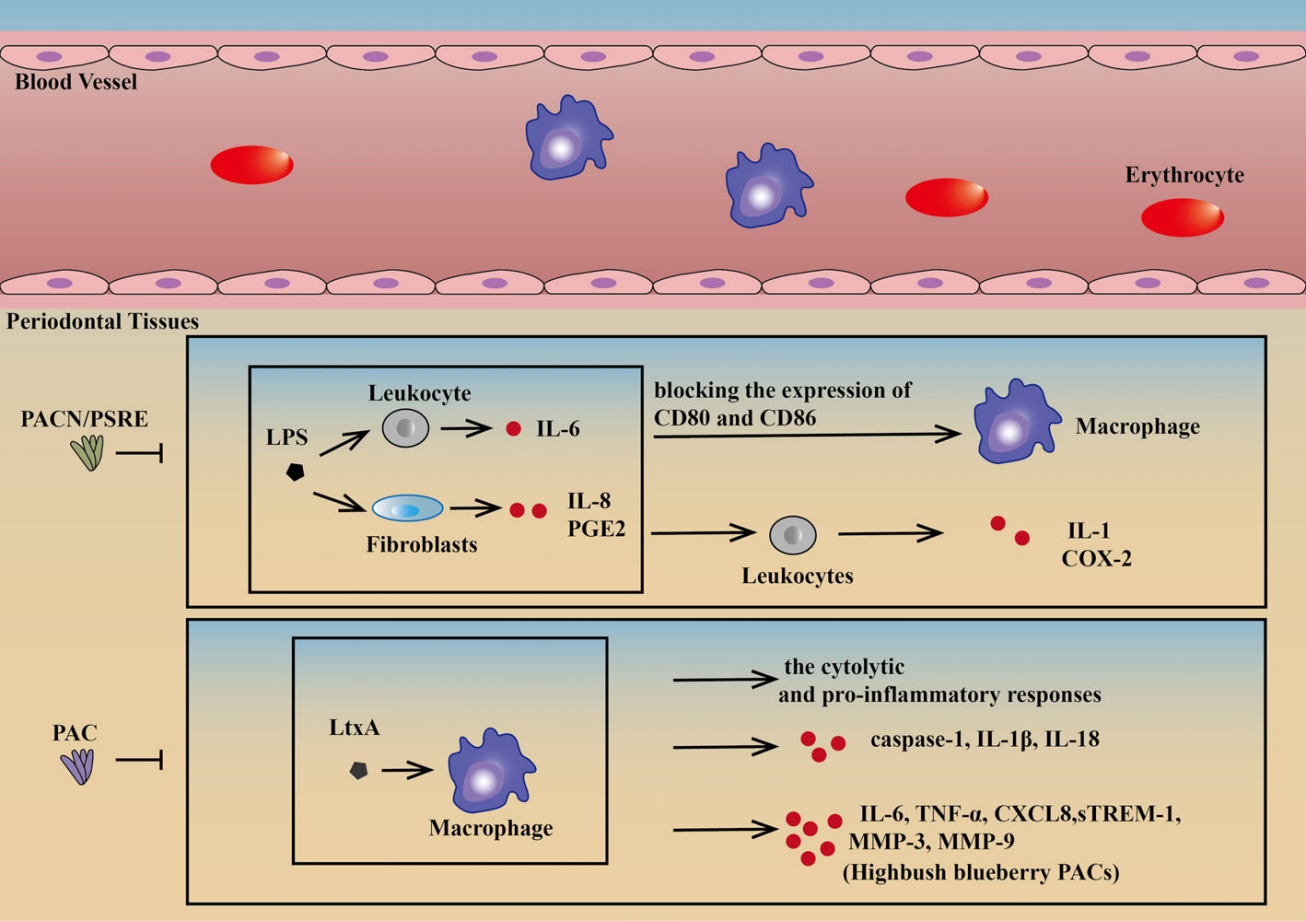
Drugs targeting macrophages. PSRE and PACN can decrease IL-8 and PGE2 by lipopolysaccharide-induced fibroblasts and IL-6 by leukocytes, blocking the expression of CD80 and CD86 on the surface of macrophages and IL-1 and COX-2 in leukocytes (64). PAC can protect macrophages against the cytotoxic effect of purified LtxA, reducing caspase-1 activation in LtxA-treated macrophages, consequently decreasing the release of IL-1β and IL-18. PACs can also neutralize the cytolytic and pro-inflammatory responses of human macrophages treated with LtxA. In addition, highbush blueberry PACs can also inhibit the secretion of IL-6, CXCL8, TNF-α, MMP-3, MMP-9, and sTREM-1in a dose-dependent manner (57, 65). PSRE, Pelargonium sidoides DC root extract; PACN, Proanthocyanidins; PAC, Proanthocyanidins.

PACs can neutralize the cytolytic and proinflammatory responses in human macrophages treated with LtxA. It can protect macrophages against the cytotoxic effect of purified LtxA, inhibiting caspase-1 activation, and consequently decreasing the secretion of IL-1β and IL-18. Apart from the above therapeutic effects, highbush blueberry PACs can also inhibit the release of TNF-α, IL-6, CXCL8, matrix metalloproteinase-3 (MMP-3), MMP-9, and TREM-1 in a dose-dependent manner. PACs have been a potential candidate for the treatment and prevention of periodontal disease because of the combination of strong pathogen-selective antibacterial, anti-inflammatory, and gingival tissue protection properties (57, 65) **(** **Figure 3** **)**.

By summarizing the drugs studied in recent years, we found that most of the drugs can affect the polarization and infiltration behaviors of macrophages [such as CSINCpi-2 (66), Metformin (67), and CCL2 MPs (68)] and inhibit the production of a variety of pro-inflammatory cytokines by macrophages [such as PMX205 (69), CSINCpi-2 (66), 6-Shogaol (70)], a few drugs can promote the production of anti-inflammatory cytokines [e.g. PMX205 (69)]. In addition, they still have some other effects, such as promoting bone regeneration [Dioscin (71)], antibacterial (Perillyl alcohol [POH) (72)] and so on. The effects of other drugs targeting macrophages with positive effects either on cell behavior or molecular expression are shown in **Table 1**.

**Table 1:** Effects of drugs targeting macrophages.

	Macrophage activity	Molecular expression	Inflammatory response	Other effects
PSRE and PACN (64)	CD80, CD86 ↓	IL-8, PGE2, IL-6, IL-1, COX-2 ↓	/	/
PAC (57, 65)	Pro-inflammatory responses ↓	IL-1β, IL-18 ↓; IL-6, CXCL8, TNF-α, MMP-3, MMP-9, sTREM-1 (highbush blueberry PACs) ↓	↓	Pathogen-selective antibacterial Gingival tissue protecting properties
Chemically-Modified Curcumin 2.24 (CMC2.24) (73, 74)	Phagocytic activity↓	TNF-α, IL-1β, IL-10, MMP-9, MMP-2 ↓; ROS ↑	/	Bone resorption↓
PMX205 (69)	Macrophage phagocytosis function ↑	NO, IL-23, TGF-β1, IL-10, Arg-1↑; Macrophage TNF-α, IL-6↓	↓	C5a receptor antagonist
CsinCPI-2 (66)	M1 Polarization↓ (Regulation of endogenous M2 macrophages)	Cathepsin K, Cathepsin B, IL-1β, TNF-α↓	↓	Caspase inhibitor
Perillyl alcohol (POH) (72)	Proliferation -	Macrophage ROS, arginase-1↓	/	Antibacterial effect
Metformin (67)	Infiltration ↓	IL-1β ↓	↓	Reduce NLRP3 inflammatory response activity by inhibiting Nek7 expression
Glyburide (75)	Infiltration ↓	Macrophage IL-1β ↓	↓	Osteoclast number ↓
Gliclazide (76)	Migration ↓	Myeloperoxidase activity, malondialdehyde, IL-1β, TNF-α, COX-2, cathepsin k, MMP-2, RANK, RANKL, SOD-1, GPx-1, MIF, PI3k, NF-kaP50, PI3k, AKT, F4/80 ↓; OPG↑	↓	Bone loss ↓
6-Shogaol (70)	Number ↓	TNF-α, IL-1β ↓	/	Active ingredients of ginger Neutrophil count↓
Tea polyphenols (77)	/	/	↓	Protect gingival keratinocytes from TNF-α-induced tight junction barrier dysfunction
Dioscin (71)	/	IL-1β, NLRP3, Caspase-1 (macrophages- derived) ↓	↓	Osteo-genesis↑
Ursodeoxycholic acid (UDCA) (78)	/	Macrophage pro-inflammatory cytokines ↓	/	/
Catechin (79)	/	Pro-IL-1β, IL-1β ↓	↓	/
An ethanol extract of paracasei NTU 101 (NTU101FM) (80)	/	Macrophage pro-inflammatory cytokines ↓	/	Antibacterial activity Osteoclast differentiation ↓
Hinokatil (81)	/	Macrophage inflammatory cytokine related gene mRNA levels ↓	/	Local treatment Alveolar bone loss, osteoclast differentiation ↓
CCL2 MPs (68)	M1 Polarization ↓ (Regulation of endogenous M2 macrophages)	/	↓	/
Triclosan (82)	/	*In vitro* protein citrullination and carbamylation of macrophages ↓; post-translational protein modification↓	/	As an adjuvant treatment for inflammatory periodontal disease

### Drugs Targeting T Lymphocytes

T lymphocytes in tissues can maintain balanced in the gingival environment (83). The number of T cells is significantly higher and they play an important role in alveolar bone resorption (84–86). The differentiation of T cells is caused by ongoing microbial challenges and the ensuing inflammation (87). The activation of different T cell subtypes controls chronic inflammation through secretion of cytokines and regulation of osteoclast production, and they play an important role in determining whether the inflammatory lesion will lead to tissue-destructive periodontitis (83). Overall, Th1 and Th17 responses increase while Th2 and Treg responses decrease in periodontitis, which can independently or interactively increase the receptor activator of NF-κB ligand (RANKL)/osteoprotegerin (OPG) ratio (88, 89). The early oral infection response is mainly attributed to pathogenic Th17 up regulation or protective Treg downregulation, and this imbalance determines the resorption of alveolar bone (90). Persistent oral *P. gingivalis* infection stimulates an initial IL-17A-based response changing into a later *de novo* Th1 response with only sporadic transdifferentiation of Th17 cells (87). As for the memory T cell subsets, a significant increase in the proportion of CD4(+)CD69(+) CD103(-) memory T cells was observed in periodontitis tissues compared with healthy gingiva (91). CD4(+) memory T cells from periodontitis tissues produced either IL-17 or IFN-γ whereas CD8(+) memory T cells produced only IFN-γ (91). In addition, during the development of periodontitis, the expression levels of IFN-γ (linked to Th cell polarization toward the Th1 cells), IL-17A, IL-17F, IL-1β, IL-6, IL-23 (linked to Th cell polarization toward the Th17 cells), TNF-α, RANKL, glucocorticoid-induced TNFR-related gene (GITR), T-bet, and GATA-3 are all highly increased (92–94). While less IL-4 (linked to Th cell polarization toward the Th2 cells), IL-10 (linked to Th cell polarization toward the Treg) and transforming growth factor-β (TGF-β) are detected in the patients with periodontitis (95). The more detail of changes of various T cell subtypes in periodontitis is summarized in **Supplementary Table 1**.

Some drugs targeting T lymphocytes can regulate the differentiation of T cells to reduce inflammation, thereby reducing bone loss and improving periodontitis. Astragaloside IV (AsIV), as one of the active ingredients in the medicinal plant *Astragalus membranaceus*, can increase peripheral blood CD4(+)T cell percentages and the CD4(+)/CD8(+) T cell ratio, while the percentage of CD8(+) T cells can be significantly reduced, as well as TNF-α, IL-1β, IL-2, IgA, and IgG. The reduction in IgA and IgG may be because the drugs target CD4(+) T cells, reducing T cell-dependent antibody responses. By this mechanism, AsIV can slow the progress of periodontitis by suppressing inflammation (96).

**Figure 4:**
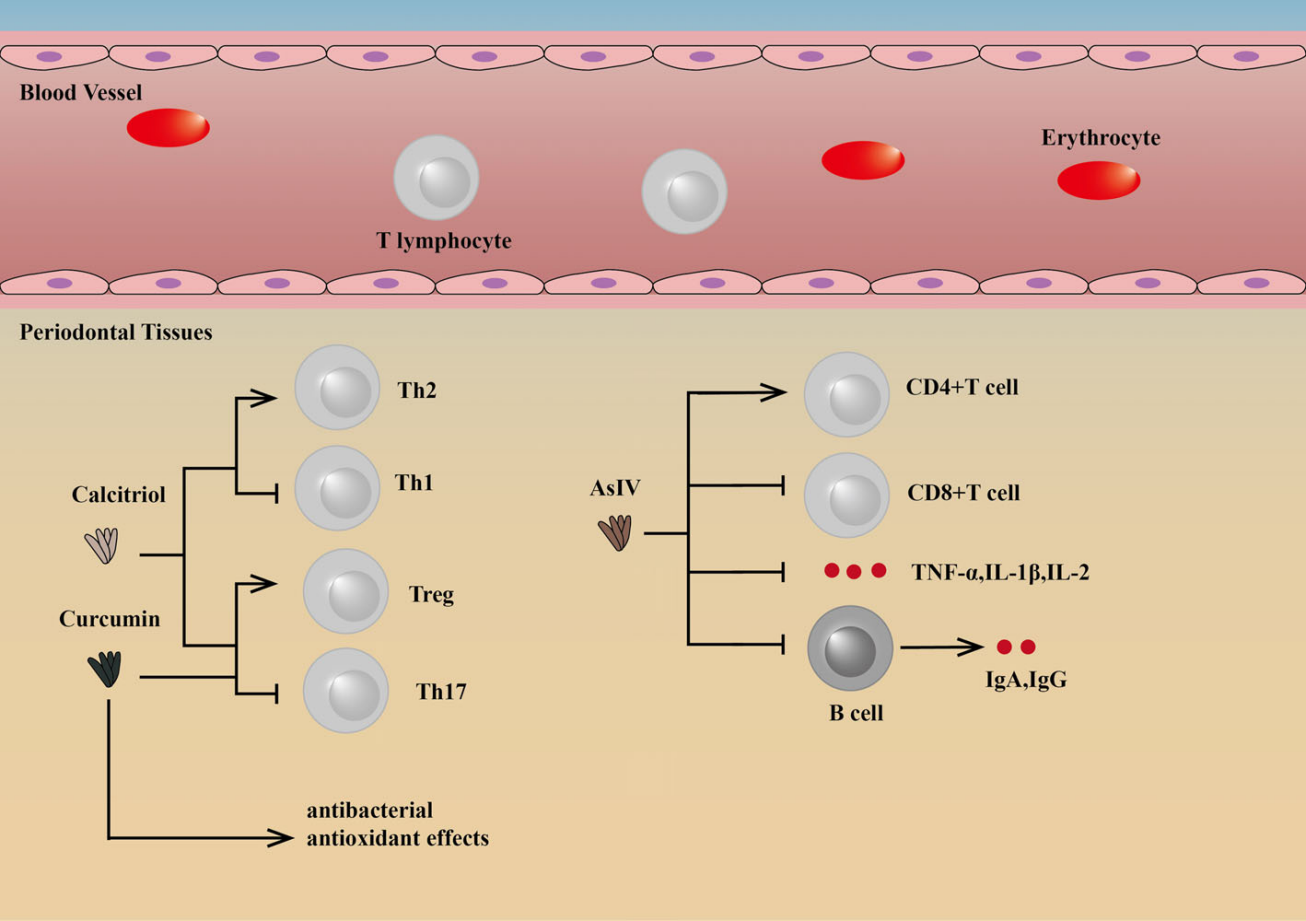
Drugs targeting T lymphocytes and their mechanisms. Curcumin and calcitriol can regulate the differentiation of Th cells, thus playing a therapeutic role. Both of them can inhibit the loss of alveolar bone by changing the proportion and function of Th cell subsets, which is manifested by the increase of Treg cells and the decrease of Th17 cells. Calcitriol intervention can also increase Th2 polarization potential and decrease Th1 promoter (97, 98). In addition, curcumin also exerts antibacterial and antioxidanteffects (99, 100). AsIV can increase peripheral blood CD4(+)T cell percentages and the CD4(+)CD8/CD8(+) T-cell ratio, while the percentage of CD8(+) T cells can be significantly reduced, as well as TNF-α, IL-1β, IL-2, IgA and IgG (96). AsIV, Astragaloside IV.

Other drugs, for example, curcumin and calcitriol, can regulate the differentiation of Th cells, thus playing a therapeutic role. Both of them can inhibit the loss of alveolar bone by changing the proportion and function of Th cell subsets, which is manifested by the increase of Tregs and the decrease of Th17 cells. Calcitriol intervention can also increase Th2 polarization potential and decrease the Th1 promoter (97, 98). In addition, curcumin also exerts antibacterial and antioxidant effects (99, 100) **(** **Figure 4** **)**.

For periodontitis in patients with Parkinson’s disease, vitamin D can reduce peripheral blood CD3, CTL counts, proinflammatory cytokines in saliva, and autophagy-related proteins in whole peripheral blood mononuclear cells. Vitamin D had varied effects on reducing systemic inflammation and promoting the induction of autophagy-related proteins related to antibacterial function. This study has entered the clinical trial stage (101).

### Drugs Targeting Cytokines

Imbalance in the inflammatory cytokine network is involved in the periodontal disease process. The interactions between the pathogens and host cells, including leukocytes, can lead to a cytokine cascade. Pro-inflammatory cytokines (such as IL-1 and TNF) lead to periodontitis while anti-inflammatory cytokines ameliorate the disease (102). At the site of periodontitis, the levels of proinflammatory cytokines such as TNF-α, IFN-γ, IL-1, IL-6, IL-12 and G-CSF are significantly increased (25, 54, 103) while anti-inflammatory cytokines are decreased (IL-4 and IL-10) (104). Cytokines and other molecules can be used to diagnose periodontitis, in which the combination of IL-6 and MMP-8 shows the best diagnostic performance (105).

Drugs that target cytokines have been mostly studied *in vitro*. They can inhibit inflammation and reduce bone resorption by inhibiting the production and action of pro-inflammatory factors. Among them, most drugs can inhibit the expression of TNF-α, such as trans-cinnamic aldehyde (106), kawa-205ME (107), β-carotene (108), calcitonin gene-related peptide (CGRP) (109), *Platycarya strobilacea* leaf extract (PLE) (110), and bismuth drugs (111). In addition, trans-cinnamic aldehyde (106), psoralen and angelicin (112), bismuth drugs (111), SIM-PPI (113), and benzydamine (114) can inhibit the expression of IL-1β. On the contrary, drugs that target cytokines have no effect on anti-inflammatory factors. This may be because some studies have shown that there is little change in anti-inflammatory cytokines in gingival crevicular fluid in patients with periodontitis (115). More detailed effects of these drugs are shown in **Table 2**.

**Table 2:** Effects of drugs targeting cytokines.

	Pro-inflammatory cytokines	Other molecules	For bone	Other effects
Trans-cinnamic aldehyde (106)	TNF-α, IL-1β ↓	/	Bone loss↓	Anti-inflammatory effects
Kava-205Me (107)	TNF-α ↓	/	/	Reduce the secretion of other cytokines involved in early inflammation, including IL-12, eotaxin, RANTES, IL-10 and IFN-γ
Carnosic Acid (116)	CXCL9, CXCL10, CXCL11 (IL-27 stimulation) ↓	/	/	By inhibiting the activation of STAT1, STAT3 and Akt
β- carotene (108)	TNF-α, IL-6, MCP-1 ↓	/	/	/
Psoralen and Angelicin (112)	IL-1β, IL-8 ↓	/	Alveolar bone loss↓	Anti-inflammatory effects
Calcitonin generelated peptide (CGRP) (109)	TNF-α (Osteoblast-derived) ↓	cCaspase3, cCaspase8 activation ↓	Osteoblast apoptosis↓	An important neuropeptide for bone remodeling
Platycarya strobilacea leaf extract (PLE) (110)	TNF-α(Macrophage-derived) ↓	MMP-9, Cathepsin K ↓	Bone resorption↓	Block NFATc1, osteoclast fusion DC-STAMP and osteoclast active cathepsin K gene expression levels
Bismuth drugs (111)	IL-6, IL-1β, TNF-α (Macrophage-derived) ↓	/	/	Anti-inflammatory effects
The Amyl-1-18 peptide (A peptide derived from rice) (117)	IL-6(Macrophage-derived)↓	/	/	Neutralize lipopolysaccharides and inhibit NF-κB signal transduction and IL-1R-related signal transduction Anti-inflammatory effects
SIM-PPi (113) Local Application of Pyrophosphorylated Simvastatin Prevents Experimental Periodontitis	IL-1β, IL-6 ↓	/	/	Synthesized by directly conjugating a SIM trimer to a pyrophosphate (PPi), greatly improving water-solubility of SIM and shows strong binding to hydroxyapatite (HA)
Benzydamine (114)	Pro-inflammatory cytokines (IL-1β) ↓	Prostaglandin↓	Bone resorption, Osteoclast differentiation↓ Osteoblast differentiation↑	Used as a cytokine inhibitor or non-steroidal anti-inflammatory drug
Flavan-3-ols and proanthocyanidins from Limonium brasiliense (118)	↓	/	/	/
Simvastatin(SIM) (113)	/	/	Osteogenesis↑	Widely used in the treatment of hypercholesterolemia HMG CoA reductase inhibitors Anti-inflammatory effects
Resolvins (119, 120)	/	/	/	Cannot suppress acute inflammation; prevent the prolongation of inflammation
Rice peptides REP9 and REP11 (121)	/	/	/	Transcriptional activity of inflammatory and osteoclast-related molecules↓

## Other Immune Cells Participating in Periodontitis

In addition to the immune system components aforementioned, other components such as DCs, mast cells (MCs) and B lymphocytes also play a role in the development of periodontitis.

DCs act as a bridge for innate and adaptive immune responses (122). The presence of DCs in periodontitis may be a sign of the severity of the lesion (123). Activated in periodontitis by the protein kinase B (AKT)/Forkhead box O1 (FoxO1) axis, DCs play both protective and destructive roles through activation of the acquired immune response (124, 125). DCs can promote Th17-specific differentiation (126) and Treg and Th17 responses (127), thereby alleviating periodontitis. However, *P. gingivalis* upregulated its short mfa1 fimbriae, leading to increased invasion of DCs, which may have negative consequences for the host (128).

MCs participate in immune regulation. In the evolution of periodontal disease there are significant dynamic alterations in migration and localization of MCs (129). There is a correlation between the number of MCs and depth of the gingival pocket (130). The significantly increased number of tryptase- T cell immunoglobulin mucin domain 1 (TIM-1) double-positive MCs had the similar tendency as the severity of periodontitis inflammation (131), especially in invasive periodontitis (132). Pro-inflammatory cytokines in MCs increase, including TNF-α (133), IL-31 (134), and TLR4 (135). Through releasing different proinflammatory cytokines, MCs can also promote leukocyte infiltration under various inflammatory states in the oral tissues (136).

B cells can aggravate the RANKL-dependent osteoclast differentiation in alveolar bone loss, leading the periodontitis (83) to a more severe extent compared with T cells (137). The proportion of memory B cells decreases in periodontitis, which shows the highest tendency to support osteoclast differentiation (137). However, there is also a class of B cells that produce IL-10, which can downregulate adaptive and innate immunity, inflammation, and autoimmunity (138). Migration of B10 cells in periodontitis resulted in increased IL-10, decreased IL-17 and RANKL, regulating local host immune response by reducing the expression of pro-inflammatory cytokines and inhibiting the local proliferation of Th17 cells (139).

It’s worth noting that a lot of drug research has focused on natural molecules like resveratrol and PACN. These natural molecules are a more promising alternative and adjunct to traditional antibiotic treatment strategies because they reduce the risk of developing resistance, short-term and long-term toxicity, adverse and side effects (64). However, many related researches failed to enter clinical application, one of the important reasons is that the biological activity, toxicity and other characteristics of natural molecules have not been scientifically and effectively explored. We don’t know yet how effective or safe they are. On the other hand, most of the relevant studies lacked the actual pathophysiological aspects of the disease (140). Nevertheless, the potential value of natural molecules in the development of periodontitis therapeutics requires a multidisciplinary research and development team to comprehensively address the actual disease challenges and patient treatment needs (141).

All in all, drug therapy, as a traditional method, has made faster progress compared with the emerging treatment methods. Most drugs target macrophages, T cells, and/or cytokines. Most of the drug studies are still *in vitro*, but there are still some drugs (such as ascorbic acid) that can be used in human periodontitis. At present, drug therapies targeting components of the immune system are still lacking and more laboratory and clinical studies are needed.

## Microbial Therapy

### Probiotic Therapy

Oral probiotics are a relatively safe and effective adjunctive treatment for periodontitis. Their complementary use has the potential to improve disease indicators and reduce the need for antibiotics (142). In order to make better use of adjuvant therapy of probiotics, we believe that it is necessary to find more correct probiotics strains, gain more recognition from patients, and focus on developing more individualized treatment plans (143).

Probiotic-assisted routine treatment of periodontitis treatment (144) may have a positive impact on the immune prognosis of patients. It has been found that GCF/MMP-8 levels were significantly reduced in patients treated with scraping and root planning (SRP) and probiotics combined (145). Significantly reduced levels of pro-inflammatory cytokines IL-1ß and IL-8 were observed in patients with generalized chronic periodontitis treated with a probiotic buccal adjuvant containing *Bifidobacterium animalis* subsp. *lactis (B. lactis)* HN019 for SRP (146), while the levels of β-defensin-3, TLR4, CD57 and CD4 were significantly increased (147). Supplementation with probiotics containing *Lactobacillus reuteri* during periodontal therapy was associated with a significant decrease in MMP-8 levels and a significant increase in matrix metalloproteinase-tissue inhibitor (TIMP-1) levels. This suggests that lozenges reduce inflammatory markers in the short term (148). Using the intestinal symbiotic *Akkermansia Muciniphila* in an experimental periodontitis model induced by *P. gingivalis*, alveolar bone loss was improved. *In vitro*, bone marrow macrophages increased IL-10 and decreased IL-12, and expressions of connective integrity markers such as integrin -β1, e-cadherin, and ZO-1 in gingival epithelial cells were also increased. This proves that *Akkermansia muciniphila* can be considered as an adjunct to periodontal therapy (149).

As an independent means of treatment, the therapeutic effect of probiotics has also been positive. Gastric administration of *Lactobacillus gasseri* SBT2055 in mice enhanced immune regulation and prevented periodontitis through intestinal immune system. The expression and secretion of TNF-α and IL-6 decreased significantly. The mRNA and peptide products of β-defensin-14 were significantly increased in the distal mucosa and intestinal tract of mice (150).

In addition, probiotics have also been found to have a preventive effect. Prophylactic administration of a combination of omega-3 and probiotics reduced alveolar bone loss and improved serum IL-1β, IL-6, and IL-10 levels (151).

Existing meta-analyses have shown that probiotics have a positive therapeutic effect on periodontitis (152). Many probiotic strains have strong aggregation ability, strong adhesion ability to oral tissue, and high antagonistic activity against oral pathogens. And they were largely free of antibiotic resistance (153). However, this treatment does not seem to be a permanent solution, as most of these probiotics originate from the external oral microenvironment and may not succeed in permanently colonizing the oral cavity. For them to continue to play an active therapeutic role, we need to develop a more appropriate frequency of administration (154). Therefore, the extraction of probiotics from the mouth of healthy people may promote the permanent colonization of probiotics and may be a more ideal treatment method (155).

### Antibacterial Therapy

There have been many achievements in antibacterial treatment of periodontitis, but there is still no clinical treatment available. As for antibacterial therapy, the main method is to induce immunity to pathogens through vaccination.

*P. gingivalis* capsular defect mutant strains cause reduced loss of alveolar bone because of non-expression of RANKL and a decrease in Th1/Th17 cytokines, Th1/Th17 lymphocytes, and osteoclasts (156). Subcutaneously vaccination with formalin-killed *P. gingivalis* can result in upregulation of Tregs through the production of IL-10 and TGF-β, downregulation of Th17 cells and IL-17A production and inhibition of lymphocyte proliferation (157). *P. gingivalis*-specific inflammatory immune response can be protected by therapeutic vaccination with a chimera (KAS2-A1) (parenteral or intraoral administration) immunogen targeting the major virulence factors of the bacterium, the gingipain proteinases. In addition, this protection is characterized by an antigen-specific IgG1 isotype antibody and Th2 response, which produced an effective therapeutic intervention that protected against *P. gingivalis*-induced periodontitis (158).

**Figure 5:**
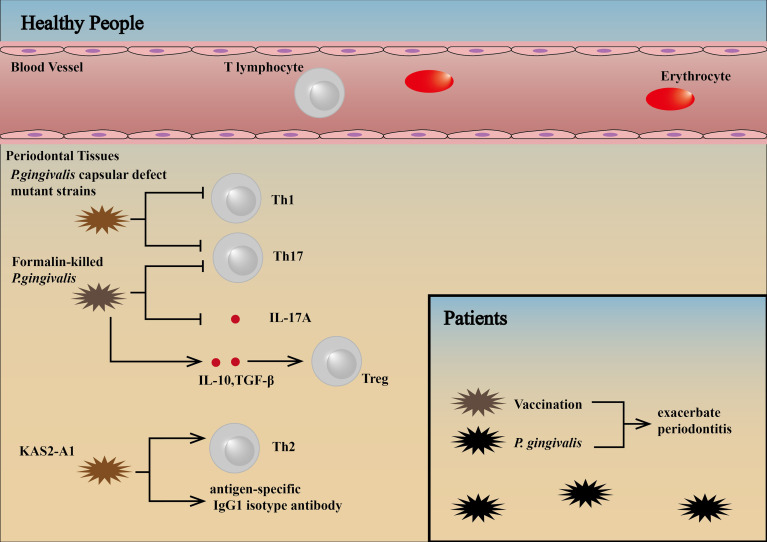
Influence of vaccination against pathogens in patients and healthy people. *P. gingivalis* capsular defect mutant strains cause reduced loss of alveolar bone because of non-expression of RANKL and a decrease in Th1/Th17 cytokines, Th1/Th17 lymphocytes, and osteoclasts (156). Subcutaneously vaccination with formalin-killed *P. gingivalis* can result in upregulation of Tregs through the production of IL-10 and TGF-β, downregulation of Th17 cells and IL-17A production and inhibition of lymphocyte proliferation (157). *P. gingivalis*-specific inflammatory immune responses can be protected by therapeutic vaccination with a chimera (KAS2-A1) immunogen targeting the major virulence factors of the bacterium, the gingipain proteinases. This protection is characterized by an antigen-specific IgG1 isotype antibody and Th2 cell response (158). Patients with *P. gingivalis*-associated periodontitis have higher threshold levels of pathogens in the subgingival plaque and exhibit an inflammatory immune response. Therefore, therapeutic vaccination may exacerbate inflammation and bone resorption in these patients (89, 94).

To date, however, *P. gingivalis* vaccination has been studied only in animals, and no effective prophylactic human periodontal vaccine has been developed, with the reason for the failure of prophylactic human periodontal vaccines unknown (157). We consider patients with *P. gingivalis*-associated periodontitis have higher threshold levels of pathogens in the subgingival plaque and exhibit an inflammatory immune response. Therefore, therapeutic vaccination may exacerbate inflammation and bone resorption in these patients **(** **Figure 5** **)**.

## Stem Cell Therapy

Mesenchymal stem cells (MSCs), including oral mesenchymal stem cells, have significant regenerative potential and immunological roles of interacting with the inflammatory microenvironment, while inflammation can also affect the properties of oral MSCs (159, 160). These cells encompass the periodontal ligament stem cells (PDLSCs), the gingival mesenchymal stem cells (GMSCs), the stem cells from human exfoliated deciduous teeth (SHED), the dental pulp stem cells (DPSCs), the dental follicle stem cells (DFSCs), the bone marrow mesenchymal stem cells (BMMSCs) and so on. With the emergence of bioengineered therapies, some studies have investigated the potential use of cell therapies, in which the main ones include undifferentiated mesenchymal cells together with different scaffolds (161). Additionally, genetic modulation may enhance the therapeutic potential of MSCs (159).

Inflamed tooth tissue, including pulp and gums, can serve as a source of MSCs with full stem cell properties. The osteogenic capability of DPSCs and GMSCs is not only preserved but increased by the overexpression of several proinflammatory cytokine-dependent chaperones and stress response proteins (162). Similarly, infected DFSCs maintain their stem cell functionality, reduce polymorphonuclear leukocyte (PMN)-induced tissue and bone degradation *via* suppression of PMN-activity, but allowed for the survival of the oral pathogens (163). However, PDLSCs from periodontitis patients are less capable of forming cell aggregates, and show impaired osteogenesis and regeneration. The decline in function can be attributed in part to TNF-α (164), which can be improved by osthole (165), tetramethylpyrazine (166) and resveratrol (167).

The immunoregulative capacity of MSCs is largely governed by the surrounding inflammatory intensity (168). Under low inflammatory condition, MSCs promote the inflammatory response through the secretion of cytokines that recruit immune cells to the local area, while if the inflammatory cytokines exceed a certain threshold, MSCs shift from pro- to anti-inflammatory cells, preventing an overexpression of immunoreaction (169–171). This means that mesenchymal stem cells can adjust their role as inflammation progresses, maintaining tissue integrity and homeostasis, which could pave the way for ameliorating periodontitis.

### PDLSCs

PDLSCs in periodontitis tissues have impaired immune regulatory function due to changes in their inflammatory microenvironment, resulting in immune response imbalance and inflammation-related bone loss. PDLSCs provide new prospects and potential therapeutic cells for tooth regeneration and reconstruction of periodontal ligament tissue damaged by periodontal disease (172, 173).

PDLSCs possess low immunogenicity and marked immunosuppression *via* PGE2-induced T-cell anergy (174). PDLSCs can induce polarization of macrophages to the M2 phenotype, which contributes to enhanced periodontal regeneration after stem cell transplantation (175). In addition, PDLSCs significantly decrease the level of non-classical major histocompatibility complex glycoprotein CD1b on DCs, resulting in defective T cell proliferation, demonstrating their potential to be utilized in promising new stem cell therapies (176). The use of allogeneic PDLSCs in a miniature pig model led to a reduction in humoral immunity. This may be because PDLSCs inhibit B cell activation through intercellular contact, mainly mediated by programmed cell death protein 1 (PD-1) and PD-L1. In addition, PDLSCs can inhibit the proliferation, differentiation and migration of B cells. But interestingly, PDLSCs enhanced B cell viability by secreting IL-6 (177).

Approaches based on extracellular vesicles (EVs) appear to provide a new paradigm for cell-free therapies that overcomes many of the clinical limitations of current cell transplantation. As an ideal vector, EVs have been shown to display anti-inflammatory and immunosuppressive actions in different tissues and could represent a potent therapeutic tools against chronic inflammation during periodontitis (178).

EVs from PDLSCs grown on gelatin-coated alginate microcarriers may be used to target chronic inflammation during periodontitis in bioreactors. EVs permanently suppressed basal and LPS-induced activity of NF-κB in PDLSCs and partially suppressed inhibitory effect of anti-TLR4 blocking Ab, without affection to osteogenic mineralization (178). MicroRNA-155-5p in PDLSC-derived EVs can upregulate sirtuin-1 in CD4(+) T cells, thereby alleviating Th17/Treg imbalance (90). Furthermore, the conditioned medium of PDLSCs reduced mRNA levels of TNF-α in periodontal healing tissue (179).

### GMSCs

GMSCs, a unique group of MSCs with the characteristic of inflammatory resistance, have been the focus of extensive research due to their easy accessibility, numerous distinct properties, including their homing to injury sites, their contribution to tissue regeneration and prominent immunomodulatory properties (160, 180).

It has been reported when transplanting GMSCs *via* the tail vein of mice, these cells were able to enter the site of periodontal injury (181). The delivery of GMSCs led to a significant decrease in TNF-α, IL-1β and IL-6 (M1 markers), a significant increase in IL-10 (M2 markers), thus inhibiting the activation of M1 macrophages (182, 183). GMSCs can also decrease the infiltration of DCs, MCs, CD8(+) T cells and Th17 cells, and increase the infiltration of Tregs (184). Hypoxic stimulation promoted the immunomodulatory properties of human GMSCs by enhancing the suppressive effects of human GMSCs on peripheral blood mononuclear cells (PBMCs) (185). In addition, GMSC-derived EVs can promote the conversion of M1 to M2 and reduce the proinflammatory cytokines produced by M1 macrophages (such as TNF-α, IL-1β and IL-12) (186).

However, many issues need to be resolved, such as costs, time-consuming culture procedures, insufficient stem cell sources, and other safety issues (187, 188).

### Other MSCs

In an induced rat model of periodontitis, SHED survived in periodontal tissue for about 7 days with minimal tissue diffusion. Then, the treatment of periodontitis with multi-dose SHED every 7 days can change the expression profile of cytokines in gingival crevicular fluid, with a reduction in the pro-inflammatory cytokines TNF-α, IFN-γ and IL-2, and an increase in the anti-inflammatory molecule IL-10. SHED can also promote the differentiation of macrophage M2 (189) and induce an immune regulatory phenotype in monocyte derived DC (moDCs) cells, thus increasing CD4(+)Foxp3(+)IL-10(+) T cells (190).

IFN-γ stimulated DFSCs by inducing immunomodulated effects of healthy donor PBMC, promoting the proliferation and differentiation of DFSCs, thereby inhibiting IL-4 and IFN-γ levels, increasing IL-10 levels, and increasing the number of CD4(+)FoxP3(+) cells (191). Dental follicle progenitor cells (DFPCs) can also sense and respond to LPS, resulting in the down-regulation of TLR4 mRNA expression and significantly increasing the migration of DFPCs. But IL-6 levels remained the same. Based on the role of DFPCs in the immune microenvironment of periodontitis, the potential of DFPCs as biological grafts for periodontal regeneration has been further confirmed (192).

Using BMMSCs in a rat model of periodontitis, significant reverse of alveolar bone lesion was observed after BMMSC transplantation. The expression of TNF-α, IFN-γ and IL-1β was down-regulated by BMMSC transplantation (193, 194). When combined with acetylsalicylic acid, the levels of TNF-α and IL-17 decreased, while the levels of IL-10 increased, and the inflammatory microenvironment was improved more (195). Injection of BMMSCs in a mouse model of periodontitis was also shown to reduce periodontitis inflammation (196). Meanwhile, BMMSCs-derived apoptotic extracellular vesicles (ApoEVs) could also regulate the polarization of macrophages (197).

DPSCs are capable of self-renewal and multidirectional differentiation, which provides a broad prospect for tooth regeneration. DPSCs have low immunogenicity and can inhibit lymphocyte proliferation and regulate cytokine production *in vitro*. DPSCs can inhibit T cell proliferation, B cell proliferation and mixed lymphocyte response. The number of Th17 cells in peripheral blood mononuclear cells co-cultured with DPSCs was significantly increased, while the number of Treg was significantly decreased. DPSCs significantly inhibited the secretion of TNF-α, IFN-γ, IL-2 and IL-17 and promoted IL-10 secretion without affecting IL-1β and IL-6 production (198, 199). These results have shed light on the therapeutic mechanism of DPSCs. In addition, DPSC-derived exosomes-incorporated chitosan hydrogel (DPSC-Exo/CS) can also facilitate macrophages to convert from a pro-inflammatory phenotype to an anti-inflammatory phenotype, thus ameliorating periodontal lesion (200).

The more detailed role of MSCs in the immune microenvironment of periodontitis is summarized in **Table 3**.

**Table 3:** Role of MSCs in anti-inflammation.

	PDLSCs	GMSCs	SHEDs	DFSCs	BMMSCs	DPSCs
cytokines	IL-6, IL-10 ↑; TNF-α, CD1b ↓	IL-10↑; TNF-α, IL-1β, IL-6, IL-12 ↓	TNF-α, IFN-γ ↓	IL-10 ↑; IFN-γ, IL-4↓	IL-10 ↑; TNF-α, IL-1α, IL-1β, IL-17, IFN-γ ↓	IL-10 ↑; TNF-α, IFN-γ, IL-2, IL-17 ↓
T lymphocytes	Anergy of T cells Imbalance of Tregs↓	Infiltration of Tregs ↑; Infiltration of CD8(+) T cells and Th17 cells↓;	TNF-α(+)IFN-γ(+)CD4(+)cells ↓	Tregs ↑	/	Tregs ↑; Th17 cells↓
B lymphocytes	Proliferation, migration, differentiation, activation of B cells ↓	/	/	/	/	/
Macrophages	M1 macrophages ↓; M2 macrophages↑	M1 macrophages ↓; M2 macrophages↑	M1 macrophages ↓; M2 macrophages↑	/	M1 macrophages ↓; M2 macrophages↑	M1 macrophages ↓; M2 macrophages↑
References	(160, 173, 175–177)	(160, 183–186)	(160, 189, 190)	(191, 192)	(194–197)	(160, 198–200)

Oral MSCs have the unique clinical advantage of availability in large numbers, controlling proliferation, migration and homing, multidirectional differentiation, and inflammatory responses. However, in order to convert laboratory periodontal regeneration methods to clinical application, the mechanisms of cell-based immunomodulatory and regeneration processes need to be understood (187).

## Gene Therapy

Gene therapy was defined as therapy “that mediates their effects by transcription and/or translation of transferred genetic material and/or by integrating into the host genome and that are administered as nucleic acids, viruses, or genetically engineered microorganisms” by the U.S. Food and Drug Administration (FDA) (201). Gene therapy has been developed to expose multiple factors to damaged surfaces for long periods of time and maintain constant protein levels, promoting recovery (202). Many studies have found that gene expression of cells in periodontitis tissues changes (203, 204). And genotypes are also important for susceptibility to periodontitis (205). These both provide the possibility of gene therapy to improve immune microenvironment, alleviating periodontitis.

Some studies have focused on immune-related genes. Through gene delivery or gene modification, it can play a good role in improving inflammation or periodontal regeneration. However, the number of studies is still inadequate and further research is needed to determine the exact effect.

It has been found that the gene modification of the P2X7 receptor (P2X7R) can promote the repair of inflammatory lesions in PDLSCs. In addition to maintaining their robust functionality under inflammatory conditions, P2X7R gene-modified stem cells may have positive influences on their neighbors through paracrine mechanism, suggesting a novel strategy to modify the harsh local microenvironment of periodontitis to accommodate stem cells and promote improved tissue regeneration (206).

Follicular dendritic cell secreted protein (FDC-SP) is considered as an immune molecule that regulates the interaction between Follicular DCs and B cells (207). FDC-SP was also found to inhibit osteogenic differentiation of human periodontal ligament cells (hPDLCs). Therefore, a stable and effective recombinant lentiviral vector expressing FDC-SP was constructed to study its effect on the phenotypic expression of hPDLCs. The results showed that in FDC-SP transfected cells, the expressions of type 1 collagen α 1, type 1 collagen α 2, and type 3 collagen were up-regulated, while the expressions of osteocalcin, osteopontin, and sialoprotein were down-regulated. In addition to the insignificant adverse effect of transfection FDC-SP on the proliferation of hPDLCs, FDC-SP can inhibit the differentiation of hPDLCs into the mineralization tissue forming cells, which can regulate the regeneration of periodontal tissue engineering (208).

Functional studies *in vitro* and *in vivo* have indicated that an isoform of Atp6i, T-cell immune response cDNA7 (TIRC7), has a significant association with the regulation of T cell and B cell activation (209). The possibility of adeno-associated virus (AAV)-mediated RNAi knockdown for the treatment of periodontal disease was first explored. AAV-small hairpin (sh) RNA-Atp6i/TIRC7 was locally injected into periodontal tissue *in vivo*, and the number of T cells in the periodontal ligament in the treatment group was significantly reduced. Meanwhile, the expression of IL-6, IL-17A, RANKL, Cathepsin K (Ctsk), acid phosphatase 5 (Acp5) and CD115 in gingival tissue was also decreased (210).

Given its crucial role and specific expression in osteoclasts, Ctsk is often considered as an important therapeutic target for targeting bone loss in periodontal disease (211). Using a known mouse model of periodontitis, AAV Expressing Ctsk shRNA (AAV-shRNA-Ctsk) was locally injected into periodontal tissues *in vivo*. AAV-shRNA-Ctsk inhibited the expression of pro-inflammatory cytokines TNF-α, INF-γ IL-1α, IL-1β, IL-12 and IL-17, but increased the expression of IL-6 in infected mice. In addition, T cells and DCs in the periodontal ligament were significantly reduced in the AAV-shRNA-Ctsk group, which significantly reduced inflammation. This suggests that AAV-mediated Ctsk silencing can significantly protect mice from *P. gingivalis* osteoclast bone resorption (212).

Injection of naked plasmid DNA encoding miR-200c into the gingiva effectively rescued miR-200c downregulation, prevented periodontal and systemic inflammation, and reduced the transcription of IL-6 and IL-8, which explained the mechanisms of gingival application of miR-200c in attenuating systemic inflammation in periodontitis (213).

Soluble protein delivery of a TNF-α antagonist inhibits alveolar bone resorption induced by periodontitis. The delivery of the TNF receptor-immunoglobulin Fc (TNFR : Fc) fusion gene to rats led to sustained therapeutic levels of serum TNFR protein and can reduce local inflammatory cell infiltration and the levels of several pro-inflammatory cytokines such as IL-1β, TNF-α, IL-6 and IL-10, protecting bone volume and density (214).

To date, most gene therapy for periodontitis has focused on bone regeneration (215–217). There is still little research on the immune microenvironment, but some of the existing studies show good application prospects (214). Gene therapy targeting the immune microenvironment can not only change the environment for cell survival but also indirectly promote bone regeneration. Transient gene expression is easier to achieve in periodontitis than in some genetic diseases that require lifelong expression of certain genes (218). At present, relatively high transfection efficiency and relatively low mutation rates can be achieved in gene therapy (219). However, the disadvantage of gene therapy is that some viral vectors themselves may induce an immune response, which may worsen the immune microenvironment of periodontitis (220). Some newly developed nonviral vectors can solve these problems to some extent (221).

## Other Therapies

The periodontal therapy (PT) associated with photodynamic therapy (PDT) reduced the expression of TNF-α in gingiva (222). Indocyanine green (ICG)-diode laser-based photothermal therapy (PTT) decreased the expression of IL-1β and MMP-8 (223). Methylene blue-mediated photodynamic therapy (MB-PDT) reduced the level of TNF-α and IL-1β and induced macrophage apoptosis through ROS and mitochondria-dependent apoptosis pathways (224). In addition, singlet phototherapy can lead to the development of reactive inflammation in periodontitis and significant vascularization of periodontal tissue, contributing to rapid tissue regeneration and stable remission (225). The above treatment methods can effectively slow down the development of periodontitis.

Low-intensity pulsed ultrasound (LIPUS) treatment inhibits the secretion of cytokines such as IL-1α, IL-1β, IL-6, IL-8, CCL2, CXCL1, and CXCL10 by periodontal ligament fibroblasts (PDLFs) and reduces the inflammatory response induced by IL-1β and TNF-α. It can also inhibit the development of periodontitis (226) **(** **Table 4** **)**.

**Table 4:** A summary of immunotherapies and their target.

	Drug therapy	Microbial therapy	Stem cell therapy	Gene therapy	Other therapy
Neutrophil	Vitamin C, 1, 25 dihydroxivitamin D3, resveratrol, quercetin, NAC	/	DFSCs, GMSCs	/	/
Monocyte	Resveratrol, metformin	/	/	/	/
Macrophage	PACN, PSRE, PACs, CMC2.24, Dioscin, Tea polyphenols, POH, 6-Shogaol, UDCA, Catechin, Metformin, Glyburide, Gliclazide, NTU101FM, Hinokatil, CCL2 MPs, CsinCPI-2, Triclosan, PMX205	/	PDLSCs, GMSCs, DPSCs, SHED, EV-GMSCs	/	MB-PDT
Lymphocyte	AsIV, Curcumin, Calcitriol, Vitamin D, antibiotic therapy	*P. gingivalis* capsular defect mutant strains, formalin-killed *P. gingivalis*, KAS2-A1	PDLSCs, GMSCs, DPSCs, SHED, DFSCs, EV-PDLSCs	TIRC7, Ctsk	/
Cytokines	Trans-cinnamic aldehyde, Resolvins, Flavan-3-ols and proanthocyanidins from Limonium Brasiliense, Benzydamine, Rice peptides REP9 and REP11, the Amyl-1-18 peptide, SIM, SIM-PPi, Kava-205Me, Carnosic Acid, β- carotene, Psoralen and Angelicin, CGRP, PLE, Bismuth drugs	*B. lactis* HN019, *Lactobacillus reuteri*, *Akkermansia muciniphila, Lactobacillus gasseri* SBT2055, *P. gingivalis* capsular defect mutant strains, formalin-killed *P. gingivalis*	PDLSCs, GMSCs, DPSCs, SHED, DFSCs, BMMSCs	P2X7R, miR-200c, TIRC7, Ctsk, TNFR: Fc	PT-PDT, ICG-PTT, MB-PDT, LIPUS

## Conclusion

The change in the immune microenvironment in periodontitis is enormous. Activity of leukocytes and inflammatory molecules increases, which can eliminate inflammation, but this excessive activity can cause great damage to the periodontal tissues, including alveolar bone. The treatment of periodontitis by modulating the immune microenvironment is a promising strategy. New anti-inflammatory and periodontal regeneration therapies can enhance the immune microenvironment and promote cell homing and tissue formation, thus achieving higher levels of immune regulation and tissue repair. In the future, more work will be needed to refine immunotherapy approaches, understand the risks and long-term efficacy of these approaches, and further develop treatment techniques to reduce the pain and social burden for patients with periodontal diseases.

## Perspectives

Great progress has been made in studying changes in the immune microenvironment of periodontitis through research on various leukocytes and cytokines that play key roles. However, as with immunotherapy for other diseases, it is necessary to research in more detail the effects of different types of immune regulation on the periodontal microenvironment and periodontal tissue regeneration, including immune response patterns and cytokine networks in periodontal tissue in both healthy and inflammatory conditions.

Pathogens induce periodontitis in susceptible patients and in most of the time the immune system is very efficient and prevents disease progression until a microbial dysbiotic environment has been established. Abundant experimental evidence shows that immunotherapy is effective in the repair and regeneration of periodontal tissue and can be used as a treatment for periodontitis. Standard therapies fail to completely solve the pathogenesis of periodontitis, but we believe that mature immune-targeted therapies will play an irreplaceable role if the immune microenvironment of periodontitis can be studied in depth. A conceptually reasonable treatment strategy for periodontitis may be the transformation of macrophages from the M1 to the M2 phenotype, increasing anti-inflammatory subtypes of T cells and anti-inflammatory cytokines and decreasing pro-inflammatory cytokines.

Previous studies have shown that it is possible to manipulate the changes in the immune microenvironment. There are many drugs that target the immune microenvironment in treating periodontitis, and they are relatively well established. The use of new therapies for anti-inflammatory and periodontal regeneration or the combination of these new approaches with existing therapeutic drugs and cytokines can enhance the generation of a viable local immune microenvironment, promote cell homing and tissue formation and thereby achieve higher levels of immune regulation and tissue repair. It is undeniable that new treatment methods have great prospects. However, they always have other defects and inappropriate places, which may be the problem of safety, the uncertainty of treatment effect, or technical and economic problems. These uncertain problems that need to be improved urgently need further research to draw scientific conclusions in order to benefit patients.

It is important to note that most of the treatments mentioned in this review have not yet been used in the clinic and cannot be used as a reference for clinical decisions. In the future, more research needs to be done to improve immunotherapy methods and understand the risks and long-term efficacy of these methods.

## Author Contributions

BY summarized the literature, provided critical comments, and wrote the manuscript. XP summarized the literature, prepared figures and wrote part of the manuscript. ZL and ZC summarized the literature and oversaw preparation of the tables and figures. YW provided critical comments and supervised all the work. All authors have made contributions to this article and approved the submitted version.

## Funding

This work was supported by National Natural Science Foundation of China (82001109, 81970975), China Postdoctoral Science Foundation (2020M683131), Guangdong Financial Fund for High-Caliber Hospital Construction (174-2018-XMZC-0001-03-0125/D-09, 174-2018-XMZC-0001-03-0125/D-10), Fundamental Research Funds for the Central Universities (20ykpy80) and Undergraduate Training Program for Innovation and Entrepreneurship of Sun Yat-sen University (20201382, 202110558092).

## Conflict of Interest

The authors declare that the research was conducted in the absence of any commercial or financial relationships that could be construed as a potential conflict of interest.

## Publisher’s Note

All claims expressed in this article are solely those of the authors and do not necessarily represent those of their affiliated organizations, or those of the publisher, the editors and the reviewers. Any product that may be evaluated in this article, or claim that may be made by its manufacturer, is not guaranteed or endorsed by the publisher.

## Supplementary Material

Supplementary Table 1 Changes of various T cell subtypes in periodontitis. MDSCs: myeloid dendritic suppressor cells; FOXO1, Forkhead box protein O1; RvD2, docosahexaenoic acid (DHA) metabolite resolvin D2; T-bet, T-cell-specific T-box transcription factor; TBX21-1993T/C (RS4794067), Tbx21 genetic polymorphism, facilitates the occurrence of Th1 immune response.

## References

[1] Hajishengallis G Korostoff JM. Revisiting the Page & Schroeder Model: The Good, the Bad and the Unknowns in the Periodontal Host Response 40 Years Later. Periodontol 2000 (2017) 75:116–51. doi: 10.1111/prd.12181 PMC5539911 28758305

[2] Hajishengallis G. Periodontitis: From Microbial Immune Subversion to Systemic Inflammation. Nat Rev Immunol (2015) 15:30–44. doi: 10.1038/nri3785 25534621 PMC4276050

[3] Offenbacher S Beck JD. Commentary: Changing Paradigms in the Oral Disease-Systemic Disease Relationship. J Periodontol (2014) 85:761–4. doi: 10.1902/jop.2014.140115 24875011

[4] Konig MF Abusleme L Reinholdt J Palmer RJ Teles RP Sampson K. Aggregatibacter Actinomycetemcomitans-Induced Hypercitrullination Links Periodontal Infection to Autoimmunity in Rheumatoid Arthritis. Sci Transl Med (2016) 8:369ra176. doi: 10.1126/scitranslmed.aaj1921 PMC5384717 27974664

[5] Lamster IB Pagan M. Periodontal Disease and the Metabolic Syndrome. Int Dent J (2017) 67:67–77. doi: 10.1111/idj.12264 27861820 PMC9376683

[6] Curtis MA Diaz PI Van Dyke TE. The Role of the Microbiota in Periodontal Disease. Periodontol 2000 (2020) 83:14–25. doi: 10.1111/prd.12296 32385883

[7] Abusleme L Dupuy AK Dutzan N Silva N Burleson JA Strausbaugh LD. The Subgingival Microbiome in Health and Periodontitis and its Relationship With Community Biomass and Inflammation. ISME J (2013) 7:1016–25. doi: 10.1038/ismej.2012.174 PMC3635234 23303375

[8] Hajishengallis G Kajikawa T Hajishengallis E Maekawa T Reis ES Mastellos DC. Complement-Dependent Mechanisms and Interventions in Periodontal Disease. Front Immunol (2019) 10:406. doi: 10.3389/fimmu.2019.00406 30915073 PMC6422998

[9] Orth RK O'Brien-Simpson NM Dashper SG Reynolds EC. Synergistic Virulence of Porphyromonas Gingivalis and Treponema Denticola in a Murine Periodontitis Model. Mol Oral Microbiol (2011) 26:229–40. doi: 10.1111/j.2041-1014.2011.00612.x 21729244

[10] Socransky SS Haffajee AD. Dental Biofilms: Difficult Therapeutic Targets. Periodontol 2000 (2002) 28:12–55. doi: 10.1034/j.1600-0757.2002.280102.x 12013340

[11] Saeki A Suzuki T Hasebe A Kamezaki R Fujita M Nakazawa F. Activation of Nucleotide-Binding Domain-Like Receptor Containing Protein 3 Inflammasome in Dendritic Cells and Macrophages by Streptococcus Sanguinis. Cell Microbiol (2017) 19:e12663. doi: 10.1111/cmi.12663 27601185

[12] Sugiyama M Saeki A Hasebe A Kamesaki R Yoshida Y Kitagawa Y. Activation of Inflammasomes in Dendritic Cells and Macrophages by Mycoplasma Salivarium. Mol Oral Microbiol (2016) 31:259–69. doi: 10.1111/omi.12117 26177301

[13] Bui FQ Johnson L Roberts J Hung SC Lee J Atanasova KR. Fusobacterium Nucleatum Infection of Gingival Epithelial Cells Leads to NLRP3 Inflammasome-Dependent Secretion of IL-1beta and the Danger Signals ASC and HMGB1. Cell Microbiol (2016) 18:970–81. doi: 10.1111/cmi.12560 PMC5101013 26687842

[14] Shenker BJ Ojcius DM Walker LP Zekavat A Scuron MD Boesze-Battaglia K. Aggregatibacter Actinomycetemcomitans Cytolethal Distending Toxin Activates the NLRP3 Inflammasome in Human Macrophages, Leading to the Release of Proinflammatory Cytokines. Infect Immun (2015) 83:1487–96. doi: 10.1128/IAI.03132-14 PMC4363449 25644004

[15] Holt SC Ebersole J Felton J Brunsvold M Kornman KS. Implantation of Bacteroides Gingivalis in Nonhuman Primates Initiates Progression of Periodontitis. Science (1988) 239:55–7. doi: 10.1126/science.3336774 3336774

[16] Socransky SS Haffajee AD Cugini MA Smith C Kent RL Jr. Microbial Complexes in Subgingival Plaque. J Clin Periodontol (1998) 25:134–44. doi: 10.1111/j.1600-051x.1998.tb02419.x 9495612

[17] Alvarez C Rojas C Rojas L Cafferata EA Monasterio G Vernal R. Regulatory T Lymphocytes in Periodontitis: A Translational View. Mediators Inflamm (2018) 2018:7806912. doi: 10.1155/2018/7806912 29805313 PMC5901475

[18] Sima C Viniegra A Glogauer M. Macrophage Immunomodulation in Chronic Osteolytic Diseases-the Case of Periodontitis. J Leukoc Biol (2019) 105:473–87. doi: 10.1002/JLB.1RU0818-310R PMC6386606 30452781

[19] Albandar JM. Aggressive and Acute Periodontal Diseases. Periodontol 2000 (2014) 65:7–12. doi: 10.1111/prd.12013 24738583

[20] Slots J. Low-Cost Periodontal Therapy. Periodontol 2000 (2012) 60:110–37. doi: 10.1111/j.1600-0757.2011.00429.x 22909110

[21] Heitz-Mayfield LJ. How Effective is Surgical Therapy Compared With Nonsurgical Debridement? Periodontol 2000 (2005) 37:72–87. doi: 10.1111/j.1600-0757.2004.03797.x 15655026

[22] Grover V Jain A Kapoor A Malhotra R Chahal GS. The Gender Bender Effect in Periodontal Immune Response. Endocr Metab Immune Disord Drug Targets (2016) 16:12–20. doi: 10.2174/1871530316666160107111301 26739959

[23] Hajishengallis G. New Developments in Neutrophil Biology and Periodontitis. Periodontol 2000 (2020) 82:78–92. doi: 10.1111/prd.12313 31850633

[24] Armstrong CL Klaes CK Vashishta A Lamont RJ Uriarte SM. Filifactor Alocis Manipulates Human Neutrophils Affecting Their Ability to Release Neutrophil Extracellular Traps Induced by PMA. Innate Immun (2018) 24:210–20. doi: 10.1177/1753425918767507 PMC6410572 29649915

[25] Zhang Z Yuan W Deng J Wang D Zhang T Peng L. Granulocyte Colony Stimulating Factor (G-CSF) Regulates Neutrophils Infiltration and Periodontal Tissue Destruction in an Experimental Periodontitis. Mol Immunol (2020) 117:110–21. doi: 10.1016/j.molimm.2019.11.003 31765840

[26] Roberts H White P Dias I McKaig S Veeramachaneni R Thakker N. Characterization of Neutrophil Function in Papillon-Lefevre Syndrome. J Leukoc Biol (2016) 100:433–44. doi: 10.1189/jlb.5A1015-489R 26957212

[27] Ustaoglu G Erdal E Inanir M. Does Periodontitis Affect Mean Platelet Volume(MPV) and Plateletcrit (PCT) Levels in Healthy Adults? Rev Assoc Med Bras (1992) (2020) 66:133–38. doi: 10.1590/1806-9282.66.2.133 32428146

[28] Hirschfeld J. Neutrophil Subsets in Periodontal Health and Disease: A Mini Review. Front Immunol (2019) 10:3001. doi: 10.3389/fimmu.2019.03001 31998301 PMC6961529

[29] Nicu EA Rijkschroeff P Wartewig E Nazmi K Loos BG. Characterization of Oral Polymorphonuclear Neutrophils in Periodontitis Patients: A Case-Control Study. BMC Oral Health (2018) 18:149. doi: 10.1186/s12903-018-0615-2 30143044 PMC6109268

[30] Rijkschroeff P Loos BG Nicu EA. Oral Polymorphonuclear Neutrophil Contributes to Oral Health. Curr Oral Health Rep (2018) 5:211–20. doi: 10.1007/s40496-018-0199-6 PMC6244624 30524928

[31] Carneiro VM Bezerra AC Guimaraes Mdo C Muniz-Junqueira MI. Decreased Phagocytic Function in Neutrophils and Monocytes From Peripheral Blood in Periodontal Disease. J Appl Oral Sci (2012) 20:503–9. doi: 10.1590/s1678-77572012000500002 PMC3881800 23138734

[32] Suarez Y Wang C Manes TD Pober JS. Cutting Edge: TNF-Induced microRNAs Regulate TNF-Induced Expression of E-Selectin and Intercellular Adhesion Molecule-1 on Human Endothelial Cells: Feedback Control of Inflammation. J Immunol (2010) 184:21–5. doi: 10.4049/jimmunol.0902369 PMC2797568 19949084

[33] Taira TM Lima V Prado DS Silva TA Issa JPM da Silva LAB. NLRP12 Attenuates Inflammatory Bone Loss in Experimental Apical Periodontitis. J Dent Res (2019) 98:476–84. doi: 10.1177/0022034518820289 30681895

[34] Magan-Fernandez A O'Valle F Abadia-Molina F Munoz R Puga-Guil P Mesa F. Characterization and Comparison of Neutrophil Extracellular Traps in Gingival Samples of Periodontitis and Gingivitis: A Pilot Study. J Periodontal Res (2019) 54:218–24. doi: 10.1111/jre.12621 30298590

[35] Ribas D Fernandez-Carranco MC Hajji N Bobadilla P Monteseirin J. Eosinophil Cationic Protein and Histamine Production by Neutrophils From Patients With Periodontitis. J Periodontol (2018) 89:228–34. doi: 10.1902/jop.2017.160679 29520824

[36] Hiyoshi T Domon H Maekawa T Nagai K Tamura H Takahashi N. Aggregatibacter Actinomycetemcomitans Induces Detachment and Death of Human Gingival Epithelial Cells and Fibroblasts *via* Elastase Release Following Leukotoxin-Dependent Neutrophil Lysis. Microbiol Immunol (2019) 63:100–10. doi: 10.1111/1348-0421.12672 30817027

[37] Zhang F Yang XM Jia SY. Characteristics of Neutrophil Extracellular Traps in Patients With Periodontitis and Gingivitis. Braz Oral Res (2020) 34:e015. doi: 10.1590/1807-3107bor-2020.vol34.0015 32130362

[38] Gu JY Liu YJ Zhu XQ Qiu JY Sun Y. Effects of Endotoxin Tolerance Induced by Porphyromonas Gingivalis Lipopolysaccharide on Inflammatory Responses in Neutrophils. Inflammation (2020) 43:1692–706. doi: 10.1007/s10753-020-01243-8 32440987

[39] Frijhoff J Winyard PG Zarkovic N Davies SS Stocker R Cheng D. Clinical Relevance of Biomarkers of Oxidative Stress. Antioxid Redox Signal (2015) 23:1144–70. doi: 10.1089/ars.2015.6317 PMC4657513 26415143

[40] Petti S Scully C. Polyphenols, Oral Health and Disease: A Review. J Dent (2009) 37:413–23. doi: 10.1016/j.jdent.2009.02.003 19303186

[41] Orihuela-Campos RC Tamaki N Mukai R Fukui M Miki K Terao J. Biological Impacts of Resveratrol, Quercetin, and N-Acetylcysteine on Oxidative Stress in Human Gingival Fibroblasts. J Clin Biochem Nutr (2015) 56:220–7. doi: 10.3164/jcbn.14-129 PMC4454086 26060353

[42] Van der Velden U. Vitamin C and Its Role in Periodontal Diseases - The Past and the Present: A Narrative Review. Oral Health Prev Dent (2020) 18:115–24. doi: 10.3290/j.ohpd.a44306 PMC11654536 32238982

[43] Staudte H Sigusch BW Glockmann E. Grapefruit Consumption Improves Vitamin C Status in Periodontitis Patients. Br Dent J (2005) 199:213–7, discussion 10. doi: 10.1038/sj.bdj.4812613 16127404

[44] Tsutsumi K Fujikawa H Kajikawa T Takedachi M Yamamoto T Murakami S. Effects of L-Ascorbic Acid 2-Phosphate Magnesium Salt on the Properties of Human Gingival Fibroblasts. J Periodontal Res (2012) 47:263–71. doi: 10.1111/j.1600-0765.2011.01430.x 22066831

[45] Tang Y Liu J Yan Y Fang H Guo C Xie R. 1,25-Dihydroxyvitamin-D3 Promotes Neutrophil Apoptosis in Periodontitis With Type 2 Diabetes Mellitus Patients *via* the P38/MAPK Pathway. Med (Baltimore) (2018) 97:e13903. doi: 10.1097/MD.0000000000013903 PMC6314780 30593206

[46] Dao VT Casas AI Maghzal GJ Seredenina T Kaludercic N Robledinos-Anton N. Pharmacology and Clinical Drug Candidates in Redox Medicine. Antioxid Redox Signal (2015) 23:1113–29. doi: 10.1089/ars.2015.6430 PMC4657508 26415051

[47] Nagasawa T Kobayashi H Aramaki M Kiji M Oda S Izumi Y. Expression of CD14, CD16 and CD45RA on Monocytes From Periodontitis Patients. J Periodontal Res (2004) 39:72–8. doi: 10.1111/j.1600-0765.2004.00713.x 14687231

[48] Almubarak A Tanagala KKK Papapanou PN Lalla E Momen-Heravi F. Disruption of Monocyte and Macrophage Homeostasis in Periodontitis. Front Immunol (2020) 11:330. doi: 10.3389/fimmu.2020.00330 32210958 PMC7067288

[49] Kumar RS Prakash S. Impaired Neutrophil and Monocyte Chemotaxis in Chronic and Aggressive Periodontitis and Effects of Periodontal Therapy. Indian J Dent Res (2012) 23:69–74. doi: 10.4103/0970-9290.99042 22842253

[50] Cheng WC van Asten SD Burns LA Evans HG Walter GJ Hashim A. Periodontitis-Associated Pathogens P. Gingivalis and A. Actinomycetemcomitans Activate Human CD14(+) Monocytes Leading to Enhanced Th17/IL-17 Responses. Eur J Immunol (2016) 46:2211–21. doi: 10.1002/eji.201545871 PMC5031191 27334899

[51] Grenier D Cazalis J Gagnon G. Response of Periodontitis and Healthy Patients in a Porphyromonas Gingivalis-Stimulated Whole-Blood Model. J Investig Clin Dent (2011) 2:38–42. doi: 10.1111/j.2041-1626.2010.00032.x 25427326

[52] Ben Lagha A Andrian E Grenier D. Resveratrol Attenuates the Pathogenic and Inflammatory Properties of Porphyromonas Gingivalis. Mol Oral Microbiol (2019) 34:118–30. doi: 10.1111/omi.12260 30950227

[53] Wang HW Lai EH Yang CN Lin SK Hong CY Yang H. Intracanal Metformin Promotes Healing of Apical Periodontitis *via* Suppressing Inducible Nitric Oxide Synthase Expression and Monocyte Recruitment. J Endod (2020) 46:65–73. doi: 10.1016/j.joen.2019.10.001 31753516

[54] Zhou LN Bi CS Gao LN An Y Chen F Chen FM. Macrophage Polarization in Human Gingival Tissue in Response to Periodontal Disease. Oral Dis (2019) 25:265–73. doi: 10.1111/odi.12983 30285304

[55] Viniegra A Goldberg H Cil C Fine N Sheikh Z Galli M. Resolving Macrophages Counter Osteolysis by Anabolic Actions on Bone Cells. J Dent Res (2018) 97:1160–69. doi: 10.1177/0022034518777973 PMC6169030 29993312

[56] Xu Z Tong Z Neelakantan P Cai Y Wei X. Enterococcus Faecalis Immunoregulates Osteoclastogenesis of Macrophages. Exp Cell Res (2018) 362:152–58. doi: 10.1016/j.yexcr.2017.11.012 29129564

[57] Ben Lagha A Howell A Grenier D. Cranberry Proanthocyanidins Neutralize the Effects of Aggregatibacter Actinomycetemcomitans Leukotoxin. Toxins (Basel) (2019) 11:662. doi: 10.3390/toxins11110662 PMC6891731 31739483

[58] Parisi L Gini E Baci D Tremolati M Fanuli M Bassani B. Macrophage Polarization in Chronic Inflammatory Diseases: Killers or Builders? J Immunol Res (2018) 2018:8917804. doi: 10.1155/2018/8917804 29507865 PMC5821995

[59] Yang J Zhu Y Duan D Wang P Xin Y Bai L. Enhanced Activity of Macrophage M1/M2 Phenotypes in Periodontitis. Arch Oral Biol (2018) 96:234–42. doi: 10.1016/j.archoralbio.2017.03.006 28351517

[60] Sun L Girnary M Wang L Jiao Y Zeng E Mercer K. IL-10 Dampens an IL-17-Mediated Periodontitis-Associated Inflammatory Network. J Immunol (2020) 204:2177–91. doi: 10.4049/jimmunol.1900532 PMC7840149 32169848

[61] Ando-Suguimoto ES Benakanakere MR Mayer MPA Kinane DF. Distinct Signaling Pathways Between Human Macrophages and Primary Gingival Epithelial Cells by Aggregatibacter Actinomycetemcomitans. Pathogens (2020) 9:248. doi: 10.3390/pathogens9040248 PMC7238148 32230992

[62] Zhong W Peng Y Yue E Huang B Zhang W Zhao Z. Gingival Crevicular Fluid Levels of SLIT3 are Increased in Periodontal Disease. Oral Dis (2020) 26:182–92. doi: 10.1111/odi.13227 31696592

[63] Bisson C Dridi SM Machouart M. Assessment of the Role of Trichomonas Tenax in the Etiopathogenesis of Human Periodontitis: A Systematic Review. PloS One (2019) 14:e0226266. doi: 10.1371/journal.pone.0226266 31846467 PMC6917263

[64] Jekabsone A Sile I Cochis A Makrecka-Kuka M Laucaityte G Makarova E. Investigation of Antibacterial and Antiinflammatory Activities of Proanthocyanidins From Pelargonium Sidoides DC Root Extract. Nutrients (2019) 11:2829. doi: 10.3390/nu11112829 PMC6893413 31752295

[65] Ben Lagha A LeBel G Grenier D. Dual Action of Highbush Blueberry Proanthocyanidins on Aggregatibacter Actinomycetemcomitans and the Host Inflammatory Response. BMC Complement Altern Med (2018) 18:10. doi: 10.1186/s12906-017-2072-x 29321009 PMC5763534

[66] Leguizamon NDP Rodrigues EM de Campos ML Nogueira AVB Viola KS Schneider VK. *In Vivo* and *In Vitro* Anti-Inflammatory and Pro-Osteogenic Effects of Citrus Cystatin CsinCPI-2. Cytokine (2019) 123:154760. doi: 10.1016/j.cyto.2019.154760 31226439

[67] Zhou X Zhang P Wang Q Ji N Xia S Ding Y. Metformin Ameliorates Experimental Diabetic Periodontitis Independently of Mammalian Target of Rapamycin (mTOR) Inhibition by Reducing NIMA-Related Kinase 7(Nek7) Expression. J Periodontol (2019) 90:1032–42. doi: 10.1002/jper.10311 30945296

[68] Zhuang Z Yoshizawa-Smith S Glowacki A Maltos K Pacheco C Shehabeldin M. Induction of M2 Macrophages Prevents Bone Loss in Murine Periodontitis Models. J Dent Res (2019) 98:200–08. doi: 10.1177/0022034518805984 PMC6761736 30392438

[69] Li G Pan J Tang Q Liu X Wang L Meng Y. Anti-Inflammatory Effects of PMX205 in Mouse Macrophage Periodontitis Model. Iran J Immunol (2018) 15:84–96 29947338 10.22034/iji.2018.39373

[70] Kim YG Kim MO Kim SH Kim HJ Pokhrel NK Lee JH. 6-Shogaol, an Active Ingredient of Ginger, Inhibits Osteoclastogenesis and Alveolar Bone Resorption in Ligature-Induced Periodontitis in Mice. J Periodontol (2019) 91:809–18. doi: 10.1002/JPER.19-0228 31675438

[71] Yin W Liu S Dong M Liu Q Shi C Bai H. A New NLRP3 Inflammasome Inhibitor, Dioscin, Promotes Osteogenesis. Small (2020) 16:e1905977. doi: 10.1002/smll.201905977 31814281

[72] Figueiredo RDA Ortega AC Gonzalez Maldonado LA Castro RD Avila-Campos MJ Rossa C. Perillyl Alcohol has Antibacterial Effects and Reduces ROS Production in Macrophages. J Appl Oral Sci (2020) 28:e20190519. doi: 10.1590/1678-7757-2019-0519 32348444 PMC7185983

[73] Deng J Golub LM Lee HM Lin MC Bhatt HD Hong HL. Chemically-Modified Curcumin 2.24: A Novel Systemic Therapy for Natural Periodontitis in Dogs. J Exp Pharmacol (2020) 12:47–60. doi: 10.2147/JEP.S236792 32104105 PMC7020920

[74] de Almeida Brandao D Spolidorio LC Johnson F Golub LM Guimaraes-Stabili MR Rossa C Jr. Dose-Response Assessment of Chemically Modified Curcumin in Experimental Periodontitis. J Periodontol (2019) 90:535–45. doi: 10.1002/JPER.18-0392 30394523

[75] Kawahara Y Kaneko T Yoshinaga Y Arita Y Nakamura K Koga C. Effects of Sulfonylureas on Periodontopathic Bacteria-Induced Inflammation. J Dent Res (2020) 99:22034520913250. doi: 10.1177/0022034520913250 32202959

[76] Araujo AA Morais HB Medeiros C Brito GAC Guedes PMM Hiyari S. Gliclazide Reduced Oxidative Stress, Inflammation, and Bone Loss in an Experimental Periodontal Disease Model. J Appl Oral Sci (2019) 27:e20180211. doi: 10.1590/1678-7757-2018-0211 30810635 PMC6382321

[77] Lagha AB Grenier D. Tea Polyphenols Protect Gingival Keratinocytes Against TNF-Alpha-Induced Tight Junction Barrier Dysfunction and Attenuate the Inflammatory Response of Monocytes/Macrophages. Cytokine (2019) 115:64–75. doi: 10.1016/j.cyto.2018.12.009 30640129

[78] Talebian R Panahipour L Gruber R. Ursodeoxycholic Acid Attenuates the Expression of Proinflammatory Cytokines in Periodontal Cells. J Periodontol (2020) 91:1098–104. doi: 10.1002/JPER.19-0013 PMC7496100 31960968

[79] Lee HA Song YR Park MH Chung HY Na HS Chung J. Catechin Ameliorates Porphyromonas Gingivalis-Induced Inflammation *via* the Regulation of TLR2/4 and Inflammasome Signaling. J Periodontol (2020) 91:661–70. doi: 10.1002/JPER.18-0004 31473995

[80] Liu TH Tsai TY Pan TM. The Anti-Periodontitis Effects of Ethanol Extract Prepared Using Lactobacillus Paracasei Subsp. Paracasei NTU 101. Nutrients (2018) 10:472. doi: 10.3390/nu10040472 PMC5946257 29649103

[81] Hiyoshi T Domon H Maekawa T Yonezawa D Kunitomo E Tabeta K. Protective Effect of Hinokitiol Against Periodontal Bone Loss in Ligature-Induced Experimental Periodontitis in Mice. Arch Oral Biol (2020) 112:104679. doi: 10.1016/j.archoralbio.2020.104679 32062102

[82] Bright R Marchant C Bartold PM. The Effect of Triclosan on Posttranslational Modification of Proteins Through Citrullination and Carbamylation. Clin Oral Investig (2018) 22:487–93. doi: 10.1007/s00784-017-2137-8 28589473

[83] Figueredo CM Lira-Junior R Love RM. T and B Cells in Periodontal Disease: New Functions in A Complex Scenario. Int J Mol Sci (2019) 20:3949. doi: 10.3390/ijms20163949 PMC6720661 31416146

[84] Thorbert-Mros S Larsson L Kalm J Berglundh T. Interleukin-17-Producing T Cells and Interleukin-17 mRNA Expression in Periodontitis and Long-Standing Gingivitis Lesions. J Periodontol (2019) 90:516–21. doi: 10.1002/JPER.18-0326 30536765

[85] Diaz-Zuniga J Melgar-Rodriguez S Monasterio G Pujol M Rojas L Alvarez C. Differential Human Th22-Lymphocyte Response Triggered by Aggregatibacter Actinomycetemcomitans Serotypes. Arch Oral Biol (2017) 78:26–33. doi: 10.1016/j.archoralbio.2017.02.008 28189882

[86] Gonzales. And B-Cell Subsets in Periodontitis. Periodontol 2000 (2015) 69:181–200. doi: 10.1111/prd.12090 26252409

[87] Bittner-Eddy PD Fischer LA Costalonga M. Transient Expression of IL-17A in Foxp3 Fate-Tracked Cells in Porphyromonas Gingivalis-Mediated Oral Dysbiosis. Front Immunol (2020) 11:677. doi: 10.3389/fimmu.2020.00677 32391008 PMC7190800

[88] Bi CS Sun LJ Qu HL Chen F Tian BM Chen FM. The Relationship Between T-Helper Cell Polarization and the RANKL/OPG Ratio in Gingival Tissues From Chronic Periodontitis Patients. Clin Exp Dent Res (2019) 5:377–88. doi: 10.1002/cre2.192 PMC7938418 31944625

[89] Stashenko P Goncalves RB Lipkin B Ficarelli A Sasaki H Campos-Neto A. Th1 Immune Response Promotes Severe Bone Resorption Caused by Porphyromonas Gingivalis. Am J Pathol (2007) 170:203–13. doi: 10.2353/ajpath.2007.060597 PMC1762702 17200194

[90] Zheng Y Dong C Yang J Jin Y Zheng W Zhou Q. Exosomal microRNA-155-5p From PDLSCs Regulated Th17/Treg Balance by Targeting Sirtuin-1 in Chronic Periodontitis. J Cell Physiol (2019) 234:20662–74. doi: 10.1002/jcp.28671 31016751

[91] Mahanonda R Champaiboon C Subbalekha K Sa-Ard-Iam N Yongyuth A Isaraphithakkul B. Memory T Cell Subsets in Healthy Gingiva and Periodontitis Tissues. J Periodontol (2018) 89:1121–30. doi: 10.1002/JPER.17-0674 29790576

[92] Alvarez C Suliman S Almarhoumi R Vega ME Rojas C Monasterio G. Regulatory T Cell Phenotype and Anti-Osteoclastogenic Function in Experimental Periodontitis. Sci Rep (2020) 10:19018. doi: 10.1038/s41598-020-76038-w 33149125 PMC7642388

[93] Sommer MEL Dalia RA Nogueira AVB Cirelli JA Vinolo MAR Fachi JL. Immune Response Mediated by Th1 / IL-17 / Caspase-9 Promotes Evolution of Periodontal Disease. Arch Oral Biol (2019) 97:77–84. doi: 10.1016/j.archoralbio.2018.09.009 30366216

[94] Moutsopoulos NM Kling HM Angelov N Jin W Palmer RJ Nares S. Porphyromonas Gingivalis Promotes Th17 Inducing Pathways in Chronic Periodontitis. J Autoimmun (2012) 39:294–303. doi: 10.1016/j.jaut.2012.03.003 22560973 PMC3416947

[95] Han YK Jin Y Miao YB Shi T Lin XP. CD8(+) Foxp3(+) T Cells Affect Alveolar Bone Homeostasis *via* Modulating Tregs/Th17 During Induced Periodontitis: An Adoptive Transfer Experiment. Inflammation (2018) 41:1791–803. doi: 10.1007/s10753-018-0822-7 29951876

[96] Zhang L Deng S. Effects of Astragaloside IV on Inflammation and Immunity in Rats With Experimental Periodontitis. Braz Oral Res (2019) 33:e032. doi: 10.1590/1807-3107bor-2019.vol33.0032 31038567

[97] Bi CS Li X Qu HL Sun LJ An Y Hong YL. Calcitriol Inhibits Osteoclastogenesis in an Inflammatory Environment by Changing the Proportion and Function of T Helper Cell Subsets (Th2/Th17). Cell Prolif (2020) 53(6):e12827. doi: 10.1111/cpr.12827 32406154 PMC7309596

[98] Bi CS Wang J Qu HL Li X Tian BM Ge S. Calcitriol Suppresses Lipopolysaccharide-Induced Alveolar Bone Damage in Rats by Regulating T Helper Cell Subset Polarization. J Periodontal Res (2019) 54:612–23. doi: 10.1111/jre.12661 31095745

[99] Izui S Sekine S Maeda K Kuboniwa M Takada A Amano A. Antibacterial Activity of Curcumin Against Periodontopathic Bacteria. J Periodontol (2016) 87:83–90. doi: 10.1902/jop.2015.150260 26447754

[100] Asteriou E Gkoutzourelas A Mavropoulos A Katsiari C Sakkas LI Bogdanos DP. Curcumin for the Management of Periodontitis and Early ACPA-Positive Rheumatoid Arthritis: Killing Two Birds With One Stone. Nutrients (2018) 10:908. doi: 10.3390/nu10070908 PMC6073415 30012973

[101] Meghil MM Hutchens L Raed A Multani NA Rajendran M Zhu H. The Influence of Vitamin D Supplementation on Local and Systemic Inflammatory Markers in Periodontitis Patients: A Pilot Study. Oral Dis (2019) 25:1403–13. doi: 10.1111/odi.13097 PMC8796207 30912231

[102] Pan W Wang Q Chen Q. The Cytokine Network Involved in the Host Immune Response to Periodontitis. Int J Oral Sci (2019) 11:30. doi: 10.1038/s41368-019-0064-z 31685798 PMC6828663

[103] Assuma R Oates T Cochran D Amar S Graves DT. IL-1 and TNF Antagonists Inhibit the Inflammatory Response and Bone Loss in Experimental Periodontitis. J Immunol (1998) 160:403–9. 9551997

[104] Bozkurt FY Yetkin Ay Z Berker E Tepe E Akkus S. Anti-Inflammatory Cytokines in Gingival Crevicular Fluid in Patients With Periodontitis and Rheumatoid Arthritis: A Preliminary Report. Cytokine (2006) 35:180–5. doi: 10.1016/j.cyto.2006.07.020 16982199

[105] Kc S Wang XZ Gallagher JE. Diagnostic Sensitivity and Specificity of Host-Derived Salivary Biomarkers in Periodontal Disease Amongst Adults: Systematic Review. J Clin Periodontol (2020) 47:289–308. doi: 10.1111/jcpe.13218 31701554

[106] Chung J Kim S Lee HA Park MH Kim S Song YR. Trans-Cinnamic Aldehyde Inhibits Aggregatibacter Actinomycetemcomitans-Induced Inflammation in THP-1-Derived Macrophages *via* Autophagy Activation. J Periodontol (2018) 89:1262–71. doi: 10.1002/JPER.17-0727 29761921

[107] Huck O Han X Mulhall H Gumenchuk I Cai B Panek J. Identification of a Kavain Analog With Efficient Anti-Inflammatory Effects. Sci Rep (2019) 9:12940. doi: 10.1038/s41598-019-49383-8 31506483 PMC6737110

[108] Kajiura Y Nishikawa Y Lew JH Kido JI Nagata T Naruishi K. Beta-Carotene Suppresses Porphyromonas Gingivalis Lipopolysaccharide-Mediated Cytokine Production in THP-1 Monocytes Cultured With High Glucose Condition. Cell Biol Int (2018) 42:105–11. doi: 10.1002/cbin.10873 28906038

[109] Zhou Y Zhang H Zhang G He Y Zhang P Sun Z. Calcitonin Generelated Peptide Reduces Porphyromonas Gingivalis LPSinduced TNFalpha Release and Apoptosis in Osteoblasts. Mol Med Rep (2018) 17:3246–54. doi: 10.3892/mmr.2017.8205 29257246

[110] Lee JH Kim H Shim JH Park J Lee SK Park KK. Platycarya Strobilacea Leaf Extract Inhibits Tumor Necrosis Factor-Alpha Production and Bone Loss Induced by Porphyromonas Gingivalis-Derived Lipopolysaccharide. Arch Oral Biol (2018) 96:46–51. doi: 10.1016/j.archoralbio.2018.08.011 30172945

[111] Cheng T Lai YT Wang C Wang Y Jiang N Li H. Bismuth Drugs Tackle Porphyromonas Gingivalis and Attune Cytokine Response in Human Cells. Metallomics (2019) 11:1207–18. doi: 10.1039/c9mt00085b 31179464

[112] Li X Yu C Hu Y Xia X Liao Y Zhang J. New Application of Psoralen and Angelicin on Periodontitis With Anti-Bacterial, Anti-Inflammatory, and Osteogenesis Effects. Front Cell Infect Microbiol (2018) 8:178. doi: 10.3389/fcimb.2018.00178 29922598 PMC5996246

[113] Chen Y Gao L Qin Q Chen S Zhang J Chen H. Comparison of 2 Different Drug-Coated Balloons in In-Stent Restenosis: The RESTORE ISR China Randomized Trial. JACC Cardiovasc Interv (2018) 11:2368–77. doi: 10.1016/j.jcin.2018.09.010 30522665

[114] Son HS Lee J Lee HI Kim N Jo YJ Lee GR. Benzydamine Inhibits Osteoclast Differentiation and Bone Resorption *via* Down-Regulation of Interleukin-1 Beta Expression. Acta Pharm Sin B (2020) 10:462–74. doi: 10.1016/j.apsb.2019.11.004 PMC7049613 32140392

[115] Cetinkaya B Guzeldemir E Ogus E Bulut S. Proinflammatory and Anti-Inflammatory Cytokines in Gingival Crevicular Fluid and Serum of Patients With Rheumatoid Arthritis and Patients With Chronic Periodontitis. J Periodontol (2013) 84:84–93. doi: 10.1902/jop.2012.110467 22414257

[116] Hosokawa I Hosokawa Y Ozaki K Matsuo T. Carnosic Acid Inhibits CXCR3 Ligands Production in IL-27-Stimulated Human Oral Epithelial Cells. Inflammation (2019) 42:1311–16. doi: 10.1007/s10753-019-00991-6 30820808

[117] Aoki-Nonaka Y Tabeta K Yokoji M Matsugishi A Matsuda Y Takahashi N. A Peptide Derived From Rice Inhibits Alveolar Bone Resorption *via* Suppression of Inflammatory Cytokine Production. J Periodontol (2019) 90:1160–69. doi: 10.1002/JPER.18-0630 31032912

[118] de Oliveira Caleare A Hensel A Mello JC Pinha AB Panizzon GP Lechtenberg M. Flavan-3-Ols and Proanthocyanidins From Limonium Brasiliense Inhibit the Adhesion of Porphyromonas Gingivalis to Epithelial Host Cells by Interaction With Gingipains. Fitoterapia (2017) 118:87–93. doi: 10.1016/j.fitote.2017.03.002 28288871

[119] Serhan CN Chiang N Van Dyke TE. Resolving Inflammation: Dual Anti-Inflammatory and Pro-Resolution Lipid Mediators. Nat Rev Immunol (2008) 8:349–61. doi: 10.1038/nri2294 PMC2744593 18437155

[120] Serhan CN. Controlling the Resolution of Acute Inflammation: A New Genus of Dual Anti-Inflammatory and Proresolving Mediators. J Periodontol (2008) 79:1520–6. doi: 10.1902/jop.2008.080231 18673006

[121] Tamura H Maekawa T Domon H Hiyoshi T Yonezawa D Nagai K. Peptides From Rice Endosperm Protein Restrain Periodontal Bone Loss in Mouse Model of Periodontitis. Arch Oral Biol (2019) 98:132–39. doi: 10.1016/j.archoralbio.2018.11.021 30485826

[122] Steinman RM. Dendritic Cells and the Control of Immunity: Enhancing the Efficiency of Antigen Presentation. Mt Sinai J Med (2001) 68:160–6. 11373688

[123] Figueiredo JAP Machado AM Oliveira VP Hartmann R Waltrick SBG Borba MG. Dendritic Cells and Their Relation to Apical Peridontitis. Braz Oral Res (2018) 32:e71. doi: 10.1590/1807-3107BOR-2018.vol32.0071 30365612

[124] Song L Dong G Guo L Graves DT. The Function of Dendritic Cells in Modulating the Host Response. Mol Oral Microbiol (2018) 33:13–21. doi: 10.1111/omi.12195 28845602 PMC5771978

[125] Xiao W Dong G Pacios S Alnammary M Barger LA Wang Y. FOXO1 Deletion Reduces Dendritic Cell Function and Enhances Susceptibility to Periodontitis. Am J Pathol (2015) 185:1085–93. doi: 10.1016/j.ajpath.2014.12.006 PMC4380851 25794707

[126] Bittner-Eddy PD Fischer LA Kaplan DH Thieu K Costalonga M. Mucosal Langerhans Cells Promote Differentiation of Th17 Cells in a Murine Model of Periodontitis But Are Not Required for Porphyromonas Gingivalis-Driven Alveolar Bone Destruction. J Immunol (2016) 197:1435–46. doi: 10.4049/jimmunol.1502693 PMC4974489 27402698

[127] Meghil MM Cutler CW. Oral Microbes and Mucosal Dendritic Cells, "Spark and Flame" of Local and Distant Inflammatory Diseases. Int J Mol Sci (2020) 21:1643. doi: 10.3390/ijms21051643 PMC7084622 32121251

[128] El-Awady A de Sousa Rabelo M Meghil MM Rajendran M Elashiry M Stadler AF. Polymicrobial Synergy Within Oral Biofilm Promotes Invasion of Dendritic Cells and Survival of Consortia Members. NPJ Biofilms Microbiomes (2019) 5:11. doi: 10.1038/s41522-019-0084-7 32179736 PMC6423025

[129] Marjanovic D Andjelkovic Z Brkic Z Videnovic G Sehalic M Matvjenko V. Quantification of Mast Cells in Different Stages of Periodontal Disease. Vojnosanit Pregl (2016) 73:458–62. doi: 10.2298/vsp141222030m 27430110

[130] ER LSF Dos Santos JN Rocha CAG Cury PR. Association Between Mast Cells and Collagen Maturation in Chronic Periodontitis in Humans. J Histochem Cytochem (2018) 66:467–75. doi: 10.1369/0022155418765131 PMC5977442 29553869

[131] Tang YC Li J Huang SG. Tryptase and TIM-1 Double-Positive Mast Cells in Different Stages of Human Chronic Periodontitis. Int J Clin Exp Pathol (2018) 11:462–71. PMC6958040 31938132

[132] Shahsavari M Azizi Mazreah S Arbabi Kalati P. Expression of Mast Cell in Aggressive Periodontitis. Minerva Stomatol (2020) 69:127–32. doi: 10.23736/S0026-4970.20.04269-7 32203646

[133] Malcolm J Millington O Millhouse E Campbell L Adrados Planell A Butcher JP. Mast Cells Contribute to Porphyromonas Gingivalis-Induced Bone Loss. J Dent Res (2016) 95:704–10. doi: 10.1177/0022034516634630 26933137

[134] Tada H Nishioka T Takase A Numazaki K Bando K Matsushita K. Porphyromonas Gingivalis Induces the Production of Interleukin-31 by Human Mast Cells, Resulting in Dysfunction of the Gingival Epithelial Barrier. Cell Microbiol (2019) 21:e12972. doi: 10.1111/cmi.12972 30423602

[135] Huang B Dai Q Huang SG. Expression of Tolllike Receptor 4 on Mast Cells in Gingival Tissues of Human Chronic Periodontitis. Mol Med Rep (2018) 17:6731–35. doi: 10.3892/mmr.2018.8648 29488617

[136] Sheethal HS Kn H Smitha T Chauhan K. Role of Mast Cells in Inflammatory and Reactive Pathologies of Pulp, Periapical Area and Periodontium. J Oral Maxillofac Pathol (2018) 22:92–7. doi: 10.4103/jomfp.JOMFP_278_17 PMC5917550 29731563

[137] Han Y Jin Y Miao Y Shi T Lin X. Improved RANKL Expression and Osteoclastogenesis Induction of CD27+CD38- Memory B Cells: A Link Between B Cells and Alveolar Bone Damage in Periodontitis. J Periodontal Res (2019) 54:73–80. doi: 10.1111/jre.12606 30346027

[138] Tedder TF. B10 Cells: A Functionally Defined Regulatory B Cell Subset. J Immunol (2015) 194:1395–401. doi: 10.4049/jimmunol.1401329 25663677

[139] Shi T Jin Y Miao Y Wang Y Zhou Y Lin X. IL-10 Secreting B Cells Regulate Periodontal Immune Response During Periodontitis. Odontology (2019) 108:350–7. doi: 10.1007/s10266-019-00470-2 31701299

[140] Jeon JG Rosalen PL Falsetta ML Koo H. Natural Products in Caries Research: Current (Limited) Knowledge, Challenges and Future Perspective. Caries Res (2011) 45:243–63. doi: 10.1159/000327250 PMC3104868 21576957

[141] Freires IA Rosalen PL. How Natural Product Research has Contributed to Oral Care Product Development? A Critical View. Pharm Res (2016) 33:1311–7. doi: 10.1007/s11095-016-1905-5 26975359

[142] Matsubara VH Bandara HM Ishikawa KH Mayer MP Samaranayake LP. The Role of Probiotic Bacteria in Managing Periodontal Disease: A Systematic Review. Expert Rev Anti Infect Ther (2016) 14:643–55. doi: 10.1080/14787210.2016.1194198 27224284

[143] Zidar A Kristl J Kocbek P Zupancic S. Treatment Challenges and Delivery Systems in Immunomodulation and Probiotic Therapies for Periodontitis. Expert Opin Drug Deliv (2021) 18:1229–44. doi: 10.1080/17425247.2021.1908260 33760648

[144] Gruner D Paris S Schwendicke F. Probiotics for Managing Caries and Periodontitis: Systematic Review and Meta-Analysis. J Dent (2016) 48:16–25. doi: 10.1016/j.jdent.2016.03.002 26965080

[145] Alshareef A Attia A Almalki M Alsharif F Melibari A Mirdad B. Effectiveness of Probiotic Lozenges in Periodontal Management of Chronic Periodontitis Patients: Clinical and Immunological Study. Eur J Dent (2020) 14:281–87. doi: 10.1055/s-0040-1709924 PMC7274828 32438428

[146] Invernici MM Salvador SL Silva PHF Soares MSM Casarin R Palioto DB. Effects of Bifidobacterium Probiotic on the Treatment of Chronic Periodontitis: A Randomized Clinical Trial. J Clin Periodontol (2018) 45:1198–210. doi: 10.1111/jcpe.12995 PMC6221043 30076613

[147] Invernici MM Furlaneto FAC Salvador SL Ouwehand AC Salminen S Mantziari A. Bifidobacterium Animalis Subsp Lactis HN019 Presents Antimicrobial Potential Against Periodontopathogens and Modulates the Immunological Response of Oral Mucosa in Periodontitis Patients. PloS One (2020) 15:e0238425. doi: 10.1371/journal.pone.0238425 32960889 PMC7508403

[148] Ince G Gursoy H Ipci SD Cakar G Emekli-Alturfan E Yilmaz S. Clinical and Biochemical Evaluation of Lozenges Containing Lactobacillus Reuteri as an Adjunct to Non-Surgical Periodontal Therapy in Chronic Periodontitis. J Periodontol (2015) 86:746–54. doi: 10.1902/jop.2015.140612 25741580

[149] Huck O Mulhall H Rubin G Kizelnik Z Iyer R Perpich JD. Akkermansia Muciniphila Reduces Porphyromonas Gingivalis-Induced Inflammation and Periodontal Bone Destruction. J Clin Periodontol (2020) 47:202–12. doi: 10.1111/jcpe.13214 31674689

[150] Kobayashi R Kobayashi T Sakai F Hosoya T Yamamoto M Kurita-Ochiai T. Oral Administration of Lactobacillus Gasseri SBT2055 is Effective in Preventing Porphyromonas Gingivalis-Accelerated Periodontal Disease. Sci Rep (2017) 7:545. doi: 10.1038/s41598-017-00623-9 28373699 PMC5428773

[151] Dogan B Kemer Dogan ES Ozmen O Fentoglu O Kirzioglu FY Calapoglu M. Synergistic Effect of Omega-3 and Probiotic Supplementation on Preventing Ligature-Induced Periodontitis. Probiotics Antimicrob Proteins (2021). doi: 10.1007/s12602-021-09803-6 34037942

[152] Martin-Cabezas R Davideau JL Tenenbaum H Huck O. Clinical Efficacy of Probiotics as an Adjunctive Therapy to non-Surgical Periodontal Treatment of Chronic Periodontitis: A Systematic Review and Meta-Analysis. J Clin Periodontol (2016) 43:520–30. doi: 10.1111/jcpe.12545 26970230

[153] Bosch M Nart J Audivert S Bonachera MA Alemany AS Fuentes MC. Isolation and Characterization of Probiotic Strains for Improving Oral Health. Arch Oral Biol (2012) 57:539–49. doi: 10.1016/j.archoralbio.2011.10.006 22054727

[154] Horz HP Meinelt A Houben B Conrads G. Distribution and Persistence of Probiotic Streptococcus Salivarius K12 in the Human Oral Cavity as Determined by Real-Time Quantitative Polymerase Chain Reaction. Oral Microbiol Immunol (2007) 22:126–30. doi: 10.1111/j.1399-302X.2007.00334.x 17311636

[155] Zupancic S Rijavec T Lapanje A Petelin M Kristl J Kocbek P. Nanofibers With Incorporated Autochthonous Bacteria as Potential Probiotics for Local Treatment of Periodontal Disease. Biomacromolecules (2018) 19:4299–306. doi: 10.1021/acs.biomac.8b01181 30289695

[156] Monasterio G Fernandez B Castillo F Rojas C Cafferata EA Rojas L. Capsular-Defective Porphyromonas Gingivalis Mutant Strains Induce Less Alveolar Bone Resorption Than W50 Wild-Type Strain Due to a Decreased Th1/Th17 Immune Response and Less Osteoclast Activity. J Periodontol (2019) 90:522–34. doi: 10.1002/JPER.18-0079 30397909

[157] Wang L Guan N Jin Y Lin X Gao H. Subcutaneous Vaccination With Porphyromonas Gingivalis Ameliorates Periodontitis by Modulating Th17/Treg Imbalance in a Murine Model. Int Immunopharmacol (2015) 25:65–73. doi: 10.1016/j.intimp.2015.01.007 25604387

[158] O'Brien-Simpson NM Holden JA Lenzo JC Tan Y Brammar GC Walsh KA. A Therapeutic Porphyromonas Gingivalis Gingipain Vaccine Induces Neutralising IgG1 Antibodies That Protect Against Experimental Periodontitis. NPJ Vaccines (2016) 1:16022. doi: 10.1038/npjvaccines.2016.22 29263860 PMC5707886

[159] Racz GZ Kadar K Foldes A Kallo K Perczel-Kovach K Keremi B. Immunomodulatory and Potential Therapeutic Role of Mesenchymal Stem Cells in Periodontitis. J Physiol Pharmacol (2014) 65:327–39. 24930504

[160] Zhou LL Liu W Wu YM Sun WL Dorfer CE Fawzy El-Sayed KM. Oral Mesenchymal Stem/Progenitor Cells: The Immunomodulatory Masters. Stem Cells Int (2020) 2020:1327405. doi: 10.1155/2020/1327405 32184830 PMC7060886

[161] Nunez J Vignoletti F Caffesse RG Sanz M. Cellular Therapy in Periodontal Regeneration. Periodontol 2000 (2019) 79:107–16. doi: 10.1111/prd.12250 30892768

[162] Tomasello L Mauceri R Coppola A Pitrone M Pizzo G Campisi G. Mesenchymal Stem Cells Derived From Inflamed Dental Pulpal and Gingival Tissue: A Potential Application for Bone Formation. Stem Cell Res Ther (2017) 8:179. doi: 10.1186/s13287-017-0633-z 28764802 PMC5540218

[163] Hieke C Kriebel K Engelmann R Muller-Hilke B Lang H Kreikemeyer B. Human Dental Stem Cells Suppress PMN Activity After Infection With the Periodontopathogens Prevotella Intermedia and Tannerella Forsythia. Sci Rep (2016) 6:39096. doi: 10.1038/srep39096 27974831 PMC5156907

[164] Zheng W Wang S Wang J Jin F. Periodontitis Promotes the Proliferation and Suppresses the Differentiation Potential of Human Periodontal Ligament Stem Cells. Int J Mol Med (2015) 36:915–22. doi: 10.3892/ijmm.2015.2314 PMC4564090 26310866

[165] Sun J Dong Z Zhang Y He X Fei D Jin F. Osthole Improves Function of Periodontitis Periodontal Ligament Stem Cells *via* Epigenetic Modification in Cell Sheets Engineering. Sci Rep (2017) 7:5254. doi: 10.1038/s41598-017-05762-7 28701802 PMC5507976

[166] Duan Y An W Wu Y Wang J. Tetramethylpyrazine Reduces Inflammation Levels and the Apoptosis of LPSstimulated Human Periodontal Ligament Cells *via* the Downregulation of Mir302b. Int J Mol Med (2020) 45:1918–26. doi: 10.3892/ijmm.2020.4554 PMC7169953 32236610

[167] Wang YJ Zhao P Sui BD Liu N Hu CH Chen J. Resveratrol Enhances the Functionality and Improves the Regeneration of Mesenchymal Stem Cell Aggregates. Exp Mol Med (2018) 50:1–15. doi: 10.1038/s12276-018-0109-y PMC6026147 29959311

[168] Wang Y Chen X Cao W Shi Y. Plasticity of Mesenchymal Stem Cells in Immunomodulation: Pathological and Therapeutic Implications. Nat Immunol (2014) 15:1009–16. doi: 10.1038/ni.3002 25329189

[169] Li W Ren G Huang Y Su J Han Y Li J. Mesenchymal Stem Cells: A Double-Edged Sword in Regulating Immune Responses. Cell Death Differ (2012) 19:1505–13. doi: 10.1038/cdd.2012.26 PMC3422473 22421969

[170] Chan JL Tang KC Patel AP Bonilla LM Pierobon N Ponzio NM. Antigen-Presenting Property of Mesenchymal Stem Cells Occurs During a Narrow Window at Low Levels of Interferon-Gamma. Blood (2006) 107:4817–24. doi: 10.1182/blood-2006-01-0057 PMC1895812 16493000

[171] Chan WK Lau AS Li JC Law HK Lau YL Chan GC. MHC Expression Kinetics and Immunogenicity of Mesenchymal Stromal Cells After Short-Term IFN-Gamma Challenge. Exp Hematol (2008) 36:1545–55. doi: 10.1016/j.exphem.2008.06.008 18715686

[172] Seo BM Miura M Gronthos S Bartold PM Batouli S Brahim J. Investigation of Multipotent Postnatal Stem Cells From Human Periodontal Ligament. Lancet (2004) 364:149–55. doi: 10.1016/S0140-6736(04)16627-0 15246727

[173] Liu Y Zheng Y Ding G Fang D Zhang C Bartold PM. Periodontal Ligament Stem Cell-Mediated Treatment for Periodontitis in Miniature Swine. Stem Cells (2008) 26:1065–73. doi: 10.1634/stemcells.2007-0734 PMC2653213 18238856

[174] Ding G Liu Y Wang W Wei F Liu D Fan Z. Allogeneic Periodontal Ligament Stem Cell Therapy for Periodontitis in Swine. Stem Cells (2010) 28:1829–38. doi: 10.1002/stem.512 PMC2996858 20979138

[175] Liu J Chen B Bao J Zhang Y Lei L Yan F. Macrophage Polarization in Periodontal Ligament Stem Cells Enhanced Periodontal Regeneration. Stem Cell Res Ther (2019) 10:320. doi: 10.1186/s13287-019-1409-4 31730019 PMC6858751

[176] Shin C Kim M Han JA Choi B Hwang D Do Y. Human Periodontal Ligament Stem Cells Suppress T-Cell Proliferation *via* Down-Regulation of non-Classical Major Histocompatibility Complex-Like Glycoprotein CD1b on Dendritic Cells. J Periodontal Res (2017) 52:135–46. doi: 10.1111/jre.12378 27021598

[177] Liu O Xu J Ding G Liu D Fan Z Zhang C. Periodontal Ligament Stem Cells Regulate B Lymphocyte Function *via* Programmed Cell Death Protein 1. Stem Cells (2013) 31:1371–82. doi: 10.1002/stem.1387 23553748

[178] Cebatariuniene A Kriauciunaite K Prunskaite J Tunaitis V Pivoriunas A. Extracellular Vesicles Suppress Basal and Lipopolysaccharide-Induced NFkappaB Activity in Human Periodontal Ligament Stem Cells. Stem Cells Dev (2019) 28:1037–49. doi: 10.1089/scd.2019.0021 31017040

[179] Onizuka S Iwata T. Application of Periodontal Ligament-Derived Multipotent Mesenchymal Stromal Cell Sheets for Periodontal Regeneration. Int J Mol Sci (2019) 20:2796. doi: 10.3390/ijms20112796 PMC6600219 31181666

[180] Fawzy El-Sayed KM Dorfer CE. Gingival Mesenchymal Stem/Progenitor Cells: A Unique Tissue Engineering Gem. Stem Cells Int (2016) 2016:7154327. doi: 10.1155/2016/7154327 27313628 PMC4903147

[181] Sun W Wang Z Xu Q Sun H Liu X Yang J. The Treatment of Systematically Transplanted Gingival Mesenchymal Stem Cells in Periodontitis in Mice. Exp Ther Med (2019) 17:2199–205. doi: 10.3892/etm.2019.7165 PMC6364184 30783482

[182] Liu X Wang Z Song W Sun W Hong R Pothukuchi A. Systematically Transplanted Human Gingiva-Derived Mesenchymal Stem Cells Regulate Lipid Metabolism and Inflammation in Hyperlipidemic Mice With Periodontitis. Exp Ther Med (2020) 19:672–82. doi: 10.3892/etm.2019.8256 PMC6913381 31885706

[183] Hong R Wang Z Sui A Liu X Fan C Lipkind S. Gingival Mesenchymal Stem Cells Attenuate Pro-Inflammatory Macrophages Stimulated With Oxidized Low-Density Lipoprotein and Modulate Lipid Metabolism. Arch Oral Biol (2019) 98:92–8. doi: 10.1016/j.archoralbio.2018.11.007 30468993

[184] Su WR Zhang QZ Shi SH Nguyen AL Le AD. Human Gingiva-Derived Mesenchymal Stromal Cells Attenuate Contact Hypersensitivity *via* Prostaglandin E2-Dependent Mechanisms. Stem Cells (2011) 29:1849–60. doi: 10.1002/stem.738 21987520

[185] Jiang CM Liu J Zhao JY Xiao L An S Gou YC. Effects of Hypoxia on the Immunomodulatory Properties of Human Gingiva-Derived Mesenchymal Stem Cells. J Dent Res (2015) 94:69–77. doi: 10.1177/0022034514557671 25403565

[186] Wang R Ji Q Meng C Liu H Fan C Lipkind S. Role of Gingival Mesenchymal Stem Cell Exosomes in Macrophage Polarization Under Inflammatory Conditions. Int Immunopharmacol (2020) 81:106030. doi: 10.1016/j.intimp.2019.106030 31796385

[187] Xu XY Li X Wang J He XT Sun HH Chen FM. Concise Review: Periodontal Tissue Regeneration Using Stem Cells: Strategies and Translational Considerations. Stem Cells Transl Med (2019) 8:392–403. doi: 10.1002/sctm.18-0181 30585445 PMC6431686

[188] Owaki T Shimizu T Yamato M Okano T. Cell Sheet Engineering for Regenerative Medicine: Current Challenges and Strategies. Biotechnol J (2014) 9:904–14. doi: 10.1002/biot.201300432 24964041

[189] Gao X Shen Z Guan M Huang Q Chen L Qin W. Immunomodulatory Role of Stem Cells From Human Exfoliated Deciduous Teeth on Periodontal Regeneration. Tissue Eng Part A (2018) 24:1341–53. doi: 10.1089/ten.TEA.2018.0016 29652608

[190] Silva Fde S Ramos RN de Almeida DC Bassi EJ Gonzales RP Miyagi SP. Mesenchymal Stem Cells Derived From Human Exfoliated Deciduous Teeth (SHEDs) Induce Immune Modulatory Profile in Monocyte-Derived Dendritic Cells. PloS One (2014) 9:e98050. doi: 10.1371/journal.pone.0098050 24846008 PMC4028272

[191] Yildirim S Zibandeh N Genc D Ozcan EM Goker K Akkoc T. The Comparison of the Immunologic Properties of Stem Cells Isolated From Human Exfoliated Deciduous Teeth, Dental Pulp, and Dental Follicles. Stem Cells Int (2016) 2016:4682875. doi: 10.1155/2016/4682875 26770205 PMC4684887

[192] Chatzivasileiou K Lux CA Steinhoff G Lang H. Dental Follicle Progenitor Cells Responses to Porphyromonas Gingivalis LPS. J Cell Mol Med (2013) 17:766–73. doi: 10.1111/jcmm.12058 PMC3823180 23560719

[193] Lu L Liu Y Zhang X Lin J. The Therapeutic Role of Bone Marrow Stem Cell Local Injection in Rat Experimental Periodontitis. J Oral Rehabil (2020) 47 Suppl 1:73–82. doi: 10.1111/joor.12843 31220354

[194] Du J Shan Z Ma P Wang S Fan Z. Allogeneic Bone Marrow Mesenchymal Stem Cell Transplantation for Periodontal Regeneration. J Dent Res (2014) 93:183–8. doi: 10.1177/0022034513513026 24226426

[195] Zhang Y Xiong Y Chen X Chen C Zhu Z Li L. Therapeutic Effect of Bone Marrow Mesenchymal Stem Cells Pretreated With Acetylsalicylic Acid on Experimental Periodontitis in Rats. Int Immunopharmacol (2018) 54:320–28. doi: 10.1016/j.intimp.2017.11.028 29195233

[196] Iguchi S Suzuki D Kawano E Mashimo T Kajiya M Toriumi T. Effect of Local Bone Marrow Stromal Cell Administration on Ligature-Induced Periodontitis in Mice. J Oral Sci (2017) 59:629–37. doi: 10.2334/josnusd.16-0033 29279573

[197] Ye QY Li ZH Wang YZ Liu SY Zhou J Liu SY. [Mesenchymal Stem Cells Derived Apoptotic Extracellular Vesicles Attenuate Pro-Inflammatory Macrophages Induced by Porphyromonas Gingivalis Lipopolysaccharide]. Zhonghua Kou Qiang Yi Xue Za Zhi (2021) 56:791–98. doi: 10.3760/cma.j.cn112144-20201027-00541 34404146

[198] Lee S Zhang QZ Karabucak B Le AD. DPSCs From Inflamed Pulp Modulate Macrophage Function *via* the TNF-Alpha/IDO Axis. J Dent Res (2016) 95:1274–81. doi: 10.1177/0022034516657817 PMC5076759 27384335

[199] Ding G Niu J Liu Y. Dental Pulp Stem Cells Suppress the Proliferation of Lymphocytes *via* Transforming Growth Factor-Beta1. Hum Cell (2015) 28:81–90. doi: 10.1007/s13577-014-0106-y 25605036

[200] Shen Z Kuang S Zhang Y Yang M Qin W Shi X. Chitosan Hydrogel Incorporated With Dental Pulp Stem Cell-Derived Exosomes Alleviates Periodontitis in Mice *via* a Macrophage-Dependent Mechanism. Bioact Mater (2020) 5:1113–26. doi: 10.1016/j.bioactmat.2020.07.002 PMC7371600 32743122

[201] Wang M Xie J Wang C Zhong D Xie L Fang H. Immunomodulatory Properties of Stem Cells in Periodontitis: Current Status and Future Prospective. Stem Cells Int (2020) 2020:9836518. doi: 10.1155/2020/9836518 32724318 PMC7366217

[202] Chen FM An Y Zhang R Zhang M. New Insights Into and Novel Applications of Release Technology for Periodontal Reconstructive Therapies. J Control Release (2011) 149:92–110. doi: 10.1016/j.jconrel.2010.10.021 20974199

[203] Cirano FR Pimentel SP Ribeiro FV Casati MZ Casarin RC Gallafassi DF. Impact of History of Periodontitis on Gene Expression of Bone-Related Factors in Young Patients. Braz Oral Res (2020) 34:e014. doi: 10.1590/1807-3107bor-2020.vol34.0014 32074214

[204] Li J Wang R Ge Y Chen D Wu B Fang F. Assessment of microRNA-144-5p and its Putative Targets in Inflamed Gingiva From Chronic Periodontitis Patients. J Periodontal Res (2019) 54:266–77. doi: 10.1111/jre.12627 30450635

[205] Brodzikowska A Gorska R Kowalski J. Interleukin-1 Genotype in Periodontitis. Arch Immunol Ther Exp (Warsz) (2019) 67:367–73. doi: 10.1007/s00005-019-00555-4 PMC6805812 31324923

[206] Xu XY Tian BM Xia Y Xia YL Li X Zhou H. Exosomes Derived From P2X7 Receptor Gene-Modified Cells Rescue Inflammation-Compromised Periodontal Ligament Stem Cells From Dysfunction. Stem Cells Transl Med (2020) 9:1414–30. doi: 10.1002/sctm.19-0418 PMC7581448 32597574

[207] Liu J Bian H Ding R Chi X Wang Y. Follicular Dendritic Cell-Secreted Protein may Enhance Osteoclastogenesis in Periodontal Disease. Connect Tissue Res (2016) 57:38–43. doi: 10.3109/03008207.2015.1095892 26577469

[208] Xiang L Ma L He Y Wei N Gong P. Transfection With Follicular Dendritic Cell Secreted Protein to Affect Phenotype Expression of Human Periodontal Ligament Cells. J Cell Biochem (2014) 115:940–8. doi: 10.1002/jcb.24736 24357406

[209] Utku N Boerner A Tomschegg A Bennai-Sanfourche F Bulwin GC Heinemann T. TIRC7 Deficiency Causes *In Vitro* and *In Vivo* Augmentation of T and B Cell Activation and Cytokine Response. J Immunol (2004) 173:2342–52. doi: 10.4049/jimmunol.173.4.2342 15294947

[210] Jiang H Chen W Zhu G Zhang L Tucker B Hao L. RNAi-Mediated Silencing of Atp6i and Atp6i Haploinsufficiency Prevents Both Bone Loss and Inflammation in a Mouse Model of Periodontal Disease. PloS One (2013) 8:e58599. doi: 10.1371/journal.pone.0058599 23577057 PMC3618217

[211] Gelb BD Shi GP Chapman HA Desnick RJ. Pycnodysostosis, a Lysosomal Disease Caused by Cathepsin K Deficiency. Science (1996) 273:1236–8. doi: 10.1126/science.273.5279.1236 8703060

[212] Chen W Gao B Hao L Zhu G Jules J MacDougall MJ. The Silencing of Cathepsin K Used in Gene Therapy for Periodontal Disease Reveals the Role of Cathepsin K in Chronic Infection and Inflammation. J Periodontal Res (2016) 51:647–60. doi: 10.1111/jre.12345 PMC5482270 26754272

[213] Krongbaramee T Zhu M Qian Q Zhang Z Eliason S Shu Y. Plasmid Encoding microRNA-200c Ameliorates Periodontitis and Systemic Inflammation in Obese Mice. Mol Ther Nucleic Acids (2021) 23:1204–16. doi: 10.1016/j.omtn.2021.01.030 PMC7899952 33664998

[214] Cirelli JA Park CH MacKool K Taba M Jr. Lustig KH Burstein H. AAV2/1-TNFR:Fc Gene Delivery Prevents Periodontal Disease Progression. Gene Ther (2009) 16:426–36. doi: 10.1038/gt.2008.174 PMC2656404 19078994

[215] Li L Jiang H Chen R Zhou J Xiao Y Zhang Y. Human Beta-Defensin 3 Gene Modification Promotes the Osteogenic Differentiation of Human Periodontal Ligament Cells and Bone Repair in Periodontitis. Int J Oral Sci (2020) 12:13. doi: 10.1038/s41368-020-0078-6 32350241 PMC7190824

[216] Yu W Zheng Y Li H Lin H Chen Z Tian Y. The Toll-Like Receptor Ligand, CpG Oligodeoxynucleotides, Regulate Proliferation and Osteogenic Differentiation of Osteoblast. J Orthop Surg Res (2020) 15:327. doi: 10.1186/s13018-020-01844-x 32795334 PMC7427903

[217] Li G Han N Yang H Zhang X Cao Y Cao Y. SFRP2 Promotes Stem Cells From Apical Papilla-Mediated Periodontal Tissue Regeneration in Miniature Pig. J Oral Rehabil (2020) 47 Suppl 1:12–8. doi: 10.1111/joor.12882 31469431

[218] Evans C. Gene Therapy for the Regeneration of Bone. Injury (2011) 42:599–604. doi: 10.1016/j.injury.2011.03.032 21489526 PMC3106986

[219] Plonka AB Khorsand B Yu N Sugai JV Salem AK Giannobile WV. Effect of Sustained PDGF Nonviral Gene Delivery on Repair of Tooth-Supporting Bone Defects. Gene Ther (2017) 24:31–9. doi: 10.1038/gt.2016.73 PMC5269540 27824330

[220] Evans CH. Gene Delivery to Bone. Adv Drug Delivery Rev (2012) 64:1331–40. doi: 10.1016/j.addr.2012.03.013 PMC3392363 22480730

[221] Chen FM Ma ZW Wang QT Wu ZF. Gene Delivery for Periodontal Tissue Engineering: Current Knowledge - Future Possibilities. Curr Gene Ther (2009) 9:248–66. doi: 10.2174/156652309788921071 19534653

[222] Murakami-Malaquias-da-Silva F Rosa EP Oliveira JG Avelar IS Palma-Cruz M Fernandes Silva JG. The Role of Periodontal Treatment Associated With Photodynamic Therapy on the Modulation of Systemic Inflammation in the Experimental Model of Asthma and Periodontitis. Photodiagnosis Photodyn Ther (2020) 29:101619. doi: 10.1016/j.pdpdt.2019.101619 31841684

[223] Chiang CP Hsieh O Tai WC Chen YJ Chang PC. Clinical Outcomes of Adjunctive Indocyanine Green-Diode Lasers Therapy for Treating Refractory Periodontitis: A Randomized Controlled Trial With *In Vitro* Assessment. J Formos Med Assoc (2020) 119:652–59. doi: 10.1016/j.jfma.2019.08.021 31543299

[224] Jiang C Yang W Wang C Qin W Ming J Zhang M. Methylene Blue-Mediated Photodynamic Therapy Induces Macrophage Apoptosis *via* ROS and Reduces Bone Resorption in Periodontitis. Oxid Med Cell Longev (2019) 2019:1529520. doi: 10.1155/2019/1529520 31485288 PMC6710739

[225] Bazikyan EA Syrnikova NV Chunikhin AA Zayratyants OV. Morphological Evaluation of Singlet Phototherapy in the Treatment of Periodontal Diseases in an Experimental Study. Stomatologiia (Mosk) (2018) 97:22–6. doi: 10.17116/stomat201897122-26 29465071

[226] Kusuyama J Nakamura T Ohnishi T Albertson BG Ebe Y Eiraku N. Low-Intensity Pulsed Ultrasound Promotes Bone Morphogenic Protein 9-Induced Osteogenesis and Suppresses Inhibitory Effects of Inflammatory Cytokines on Cellular Responses *via* Rho-Associated Kinase 1 in Human Periodontal Ligament Fibroblasts. J Cell Biochem (2019) 120:14657–69. doi: 10.1002/jcb.28727 31006911

