# Supplementary material for: Immunomodulation in the Treatment of Periodontitis: Progress and Perspectives
Source: Front Immunol. 2021 Nov 19;12:781378. doi: 10.3389/fimmu.2021.781378 (PMC8640126; doi:10.3389/fimmu.2021.781378)
Supplement: Supplementary Table 1 — Changes of various T cell subtypes in periodontitis. MDSCs: myeloid dendritic suppressor cells; FOXO1, Forkhead box protein O1; RvD2, docosahexaenoic acid (DHA) metabolite resolvin D2; T-bet, T-cell-specific T-box transcription factor; TBX21-1993T/C (RS4794067), Tbx21 genetic polymorphism, facilitates the occurrence of Th1 immune response. [file Table_1.docx]

**Supplementary Table 1.** Changes of various T cell subtypes in periodontitis.

| **Subtypes** | **Th1 cells** | **Th2 cells** | **Th17 cells** | **Tregs** | **CD8**(+)**Tregs** |
| --- | --- | --- | --- | --- | --- |
| **Number** | Increase | Decrease  (Increase) | Increase | Decrease  (Increase) | No change |
| **Inducing factor** | Serotype B *A. actinomycetemcomitans* | / | serotype B *A. actinomycetemcomitans*, Pg-LPS, switched memory B cell | MDSCs  FOXO1 | / |
| **inhibitory factor** | RvD2 | / | *T. forsythia*-specific glycan portion | / | / |
| **Features** | TBX21-1993T /C (RS4794067) | / | Dependent on the local probiotics, requiring IL-6 and IL-23 | / | / |
| **Molecular expression** | IFN-γ, TNF-α, IL-1β, IL-18, T-bet | IFN-γ, TGF-β, IL-4 | RANKL, OPG, IL-6, TNF-α, IFN-γ, IL-1β, IL-17A | IL-10 and TGF-β | IL-1β、IL-6、IL-17A、RANKL |
| **Result** | Promote periodontitis | Inflammation lessens when Th2 cells assist T cells  Osteoprotective role in periodontitis | Bacterial invasion leads to the generation of specialized TH17 cells which may have developed to stop local infection by inducing tooth loss, function as a double-edged sword by protecting against pathogens while also inducing skeletal tissue degradation | Protect periodontal tissue from inflammatory destruction and play an active regulatory role in periodontitis;  Play a more prominent role in periodontitis than Th17 cells | Affect the homeostasis of alveolar bone by regulating Tregs/Th17 cells;  Reduce the above lesions;  Reduce alveolar bone destruction and osteoclast formation |
| **Reference** | (1-6) | (3, 7, 8) | (4, 5, 9-15) | (15-21) | (15) |

MDSCs: myeloid dendritic suppressor cells; FOXO1: Forkhead box protein O1; RvD2: docosahexaenoic acid (DHA) metabolite resolvin D2; T-bet: T-cell-specific T-box transcription factor; TBX21-1993T /C (RS4794067): Tbx21 genetic polymorphism, facilitates the occurrence of Th1 immune response

References:

1. Mizraji G, Heyman O, Van Dyke TE, Wilensky A. Resolvin D2 Restrains Th1 Immunity and Prevents Alveolar Bone Loss in Murine Periodontitis. *Front Immunol*. (2018) 9:785. doi: 10.3389/fimmu.2018.00785

2. Colavite PM, Cavalla F, Garlet TP, Azevedo MCS, Melchiades JL, Campanelli AP, et al. TBX21-1993T/C polymorphism association with Th1 and Th17 response at periapex and with periapical lesions development risk. *J Leukoc Biol*. (2019) 105:609-19. doi: 10.1002/JLB.6A0918-339R

3. Colic M, Lukic A, Vucevic D, Milosavljevic P, Majstorovic I, Marjanovic M, et al. Correlation between phenotypic characteristics of mononuclear cells isolated from human periapical lesions and their in vitro production of Th1 and Th2 cytokines. *Arch Oral Biol*. (2006) 51:1120-30. doi: 10.1016/j.archoralbio.2006.05.003

4. Monasterio G, Castillo F, Ibarra JP, Guevara J, Rojas L, Alvarez C, et al. Alveolar bone resorption and Th1/Th17-associated immune response triggered during Aggregatibacter actinomycetemcomitans-induced experimental periodontitis are serotype-dependent. *J Periodontol*. (2018) 89:1249-61. doi: 10.1002/JPER.17-0563

5. Chen XT, Chen LL, Tan JY, Shi DH, Ke T, Lei LH. Th17 and Th1 Lymphocytes Are Correlated with Chronic Periodontitis. *Immunol Invest*. (2016) 45:243-54. doi: 10.3109/08820139.2016.1138967

6. Orozco A, Gemmell E, Bickel M, Seymour GJ. Interleukin-1beta, interleukin-12 and interleukin-18 levels in gingival fluid and serum of patients with gingivitis and periodontitis. *Oral Microbiol Immunol*. (2006) 21:256-60. doi: 10.1111/j.1399-302X.2006.00292.x

7. Beklen A. Effects of IL-13 on TGF-beta and MMP-1 in periodontitis. *Biotech Histochem*. (2017) 92:374-80. doi: 10.1080/10520295.2017.1312526

8. Behfarnia P, Birang R, Pishva SS, Hakemi MG, Khorasani MM. Expression levels of th-2 and th-17 characteristic genes in healthy tissue versus periodontitis. *J Dent (Tehran)*. (2013) 10:23-31.

9. Tsukasaki M, Komatsu N, Nagashima K, Nitta T, Pluemsakunthai W, Shukunami C, et al. Host defense against oral microbiota by bone-damaging T cells. *Nat Commun*. (2018) 9:701. doi: 10.1038/s41467-018-03147-6

10. Zhang L, Gao L, Xu C, Li X, Wang P, Zhang C, et al. Porphyromonas gingivalis lipopolysaccharide promotes T- helper 17 cell differentiation from human CD4(+) naive T cells via toll-like receptor-2 in vitro. *Arch Oral Biol*. (2019) 107:104483. doi: 10.1016/j.archoralbio.2019.104483

11. Tomek MB, Maresch D, Windwarder M, Friedrich V, Janesch B, Fuchs K, et al. A General Protein O-Glycosylation Gene Cluster Encodes the Species-Specific Glycan of the Oral Pathogen Tannerella forsythia: O-Glycan Biosynthesis and Immunological Implications. *Front Microbiol*. (2018) 9:2008. doi: 10.3389/fmicb.2018.02008

12. Gaffen SL, Moutsopoulos NM. Regulation of host-microbe interactions at oral mucosal barriers by type 17 immunity. *Sci Immunol*. (2020) 5:doi: 10.1126/sciimmunol.aau4594

13. Dutzan N, Kajikawa T, Abusleme L, Greenwell-Wild T, Zuazo CE, Ikeuchi T, et al. A dysbiotic microbiome triggers TH17 cells to mediate oral mucosal immunopathology in mice and humans. *Sci Transl Med*. (2018) 10:doi: 10.1126/scitranslmed.aat0797

14. Han Y, Jin Y, Miao Y, Shi T, Lin X. Switched memory B cells promote alveolar bone damage during periodontitis: An adoptive transfer experiment. *Int Immunopharmacol*. (2018) 62:147-54. doi: 10.1016/j.intimp.2018.07.003

15. Han YK, Jin Y, Miao YB, Shi T, Lin XP. CD8(+) Foxp3(+) T Cells Affect Alveolar Bone Homeostasis via Modulating Tregs/Th17 During Induced Periodontitis: an Adoptive Transfer Experiment. *Inflammation*. (2018) 41:1791-803. doi: 10.1007/s10753-018-0822-7

16. Arjunan P, Meghil MM, Pi W, Xu J, Lang L, El-Awady A, et al. Oral Pathobiont Activates Anti-Apoptotic Pathway, Promoting both Immune Suppression and Oncogenic Cell Proliferation. *Sci Rep*. (2018) 8:16607. doi: 10.1038/s41598-018-35126-8

17. Graves DT, Milovanova TN. Mucosal Immunity and the FOXO1 Transcription Factors. *Front Immunol*. (2019) 10:2530. doi: 10.3389/fimmu.2019.02530

18. Nakajima T, Ueki-Maruyama K, Oda T, Ohsawa Y, Ito H, Seymour GJ, et al. Regulatory T-cells infiltrate periodontal disease tissues. *J Dent Res*. (2005) 84:639-43. doi: 10.1177/154405910508400711

19. da Motta RJG, Almeida LY, Villafuerte KRV, Ribeiro-Silva A, Leon JE, Tirapelli C. FOXP3+ and CD25+ cells are reduced in patients with stage IV, grade C periodontitis: A comparative clinical study. *J Periodontal Res*. (2019) doi: 10.1111/jre.12721

20. Arul D, Rao S. Isolation of Naturally Induced T-regulatory Cells in Gingival Tissues of Healthy Human Subjects and Subjects with Gingivitis and Chronic Periodontitis. *Cureus*. (2019) 11:e4283. doi: 10.7759/cureus.4283

21. Parachuru VPB, Coates DE, Milne TJ, Rich AM, Seymour GJ. FoxP3(+) regulatory T cells, interleukin 17 and mast cells in chronic inflammatory periodontal disease. *J Periodontal Res*. (2018) 53:622-35. doi: 10.1111/jre.12552
